# Inferring cell-type-specific causal gene regulatory networks during human neurogenesis

**DOI:** 10.1186/s13059-023-02959-0

**Published:** 2023-05-30

**Authors:** Nil Aygün, Dan Liang, Wesley L. Crouse, Gregory R. Keele, Michael I. Love, Jason L. Stein

**Affiliations:** 1 Department of Genetics, University of North Carolina at Chapel Hill, Chapel Hill, NC 27599 USA; 2 UNC Neuroscience Center, University of North Carolina at Chapel Hill, Chapel Hill, NC 27599 USA; 3 Department of Human Genetics, University of Chicago, Chicago, IL 60637 USA; 4 The Jackson Laboratory, 600 Main Street, Bar Harbor, ME 04609 USA; 5 Department of Biostatistics, University of North Carolina at Chapel Hill, Chapel Hill, NC 27599 USA

**Keywords:** Causal inference, Gene regulatory networks, Quantitative trait loci, Cis- and trans-regulation, Neurogenesis

## Abstract

**Background:**

Genetic variation influences both chromatin accessibility, assessed in chromatin accessibility quantitative trait loci (caQTL) studies, and gene expression, assessed in expression QTL (eQTL) studies. Genetic variants can impact either nearby genes (cis-eQTLs) or distal genes (trans-eQTLs). Colocalization between caQTL and eQTL, or cis- and trans-eQTLs suggests that they share causal variants. However, pairwise colocalization between these molecular QTLs does not guarantee a causal relationship. Mediation analysis can be applied to assess the evidence supporting causality versus independence between molecular QTLs. Given that the function of QTLs can be cell-type-specific, we performed mediation analyses to find epigenetic and distal regulatory causal pathways for genes within two major cell types of the developing human cortex, progenitors and neurons.

**Results:**

We find that the expression of 168 and 38 genes is mediated by chromatin accessibility in progenitors and neurons, respectively. We also find that the expression of 11 and 12 downstream genes is mediated by upstream genes in progenitors and neurons. Moreover, we discover that a genetic locus associated with inter-individual differences in brain structure shows evidence for mediation of SLC26A7 through chromatin accessibility, identifying molecular mechanisms of a common variant association to a brain trait.

**Conclusions:**

In this study, we identify cell-type-specific causal gene regulatory networks whereby the impacts of variants on gene expression were mediated by chromatin accessibility or distal gene expression. Identification of these causal paths will enable identifying and prioritizing actionable regulatory targets perturbing these key processes during neurodevelopment.

**Supplementary Information:**

The online version contains supplementary material available at 10.1186/s13059-023-02959-0.

## Background

**Fig. 1:**
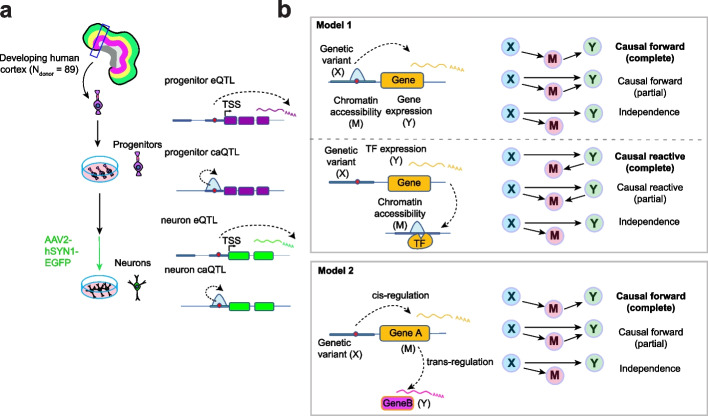
Study design. **a** Cell-type-specific in vitro experimental system including progenitors and their differentiated neuronal progeny. **b** Models evaluated in this study

Genome-wide association studies (GWAS) have identified many common genetic variants associated with risk for neuropsychiatric disorders [1–3] and inter-individual differences in other brain-relevant traits, like cortical structure [4–6]. GWAS studies alone do not yield molecular, cellular, and systems-level causal pathways by which discovered genetic variation influences a trait. Given the enrichment of brain trait-associated variants within non-coding regulatory elements [7, 8], quantitative trait loci (QTL) analyses for gene regulatory phenotypes, including chromatin accessibility and gene expression, have been widely applied for the functional interpretation of GWAS (Fig. 1a) [9–12]. Importantly, recent QTL studies have demonstrated that these non-coding brain trait-associated variants exert their functional effects in a developmental and cell-type-specific manner [13–16].

To find the causal gene regulatory networks by which non-coding genetic variation influences a complex brain or behavioral trait, first, a colocalization between a molecular QTL, performed in a relevant cell type and developmental time period, and the trait GWAS is conducted to identify shared causal variants [11, 17]. Similarly, colocalization between multiple molecular QTLs can be used to determine if there are shared genetic influences on both traits, for example, chromatin accessibility and gene expression [18, 19]. However, colocalization does not guarantee a causal relationship. If a genetic variant, represented by X, is significantly associated with both a candidate mediator (M) such as chromatin accessibility, and an outcome (Y) such as gene expression measured from the same individuals; X (or an LD proxy of X) can directly regulate Y independent from M, termed independence (X—> M; X—> Y); the effect of X on Y can be mediated partially or completely through M, termed a forward model (X—> M—> Y); or the effect of X on M can be partially or completely mediated by Y, termed a reactive model (X—> Y—> M) [20–22] (Fig. 1b). The causal ordering of events in each model is not dependent on the directionality of the effect of X on M or Y, which is fixed from X to M or Y. A causal pathway of a brain trait-associated variant can be used to prioritize actionable trait-relevant therapeutic targets that interrupt the key pathological processes [23–25].

Causal pathways for non-coding trait-associated genetic variation have been experimentally demonstrated for a small subset of GWAS traits. An example of the causal forward model (X—> M—> Y) is a common genetic variant (X) located within a regulatory element (M), such as a gene promoter or enhancer, that disrupts the binding motif of a transcription factor (TF) leading to differential chromatin accessibility observed via chromatin accessibility QTL (caQTL) analysis, which leads to differential expression (Y) observed via cis-expression QTL (cis-eQTL) analysis [7, 26–29]. Though less canonical, causal reactive models (X—> Y—> M) have also been experimentally verified, whereby changes in the expression of a gene encoding a TF may cause changes in the chromatin accessibility in the genomic regions harboring a TF binding motif [30, 31]. Similar gene regulatory mechanisms can lead to trans-eQTLs, whereby a genetic variant (X) cis-regulates a transcription factor or components of a signaling cascade (M) and leads to altered expression of a distal gene (Y). Alternatively, in the independence model, the expression of the gene is independent of chromatin accessibility, which may occur due to false positive colocalizations or non-canonical regulatory mechanisms [30, 32–36].

There are two common ways to test causal models using genetic association data. Mendelian randomization (MR) analyses infer causal relationships by defining “instruments”, variants that influence M and satisfy certain modeling assumptions, and determining if these instruments may affect Y through M by examining their paired effect sizes and standard errors [37–39]. MR analyses have the advantages of using summary statistics rather than raw data, and additionally, two phenotypes M and Y are not required to be measured in the same individuals. However, MR approaches require allelic heterogeneity, which for caQTLs is rarely found and for eQTLs is generally only detectable with large sample sizes [12, 13, 40]. Alternatively, mediation analysis can be performed to distinguish between pleiotropic, forward, and causal reactive models when multiple phenotypes are measured across the same donors and there is access to the raw data. Evidence for the forward model is established in a classical mediation approach when there is no uncontrolled confounding and (1) M and Y are conditionally dependent given X, and (2) X and Y are conditionally independent given M [41, 42]. Mediation approaches have been applied to individual-level multi-modal QTL data to infer causal relationships between different molecular intermediates including mediation of gene expression via DNA methylation, histone acetylation [43], chromatin accessibility [44, 45], and trans-regulation by distal genes [46–50]. However, these previous studies were subject to difficulties with the specification of multiple null hypotheses, correction for multiple testing, and quantifying evidence for complete versus partial mediation. A recent Bayesian model selection framework can overcome these challenges by weighing the evidence of each potential relationship across X-M-Y triplets, summarized as a posterior probability [22].

Current studies have investigated genetically mediated causal gene regulatory networks in the human brain using multi-modal QTLs, but they were limited to studying data derived from bulk adult brain tissue and were not able to resolve cell-type and developmentally specific mechanisms [43, 51–53]. In this study, we applied a Bayesian causal inference method, *bmediatR*, [22] to examine the mediation of genetic effects (1) on gene expression through nearby chromatin accessibility, (2) on distal chromatin accessibility through the expression of TFs, and (3) on downstream gene expression through trans-regulation by other genes using our previously generated ca/eQTL datasets derived from human cortical progenitors and their differentiated neuronal progeny (Fig. 1a) [13, 14]. We identified causal paths for gene expression mediated by chromatin accessibility for 168 and 38 genes in progenitors and neurons. Also, we found that the expression of 11 and 12 downstream genes was mediated by upstream genes proximal to cis-regulatory SNPs in progenitors and neurons, respectively. Furthermore, we proposed causal mechanisms affecting brain structure and neuropsychiatric disorders through multiple levels of biology by defining a causal forward regulatory mechanism leading to changes in expression of the *SLC26A7* gene via chromatin accessibility at a locus co-localized with middle temporal gyrus surface area GWAS [4].

## Results

### *Cell-type-specific mediation of expression *via* chromatin accessibility (forward model)*

We assessed causal mediation using previously generated cell-type specific ATAC-sequencing [13] and RNA-sequencing [14] data that was subset to a dataset where both data modalities were acquired in the same donors for each cell type (*N*_donor_ = 75 in progenitors and *N*_donor_ = 57 in neurons). To identify causal models explaining X-M-Y triplets, we identified variants impacting both chromatin accessibility and gene expression (Fig. 1b, Model 1). We first subset the ca/eQTLs to biologically relevant X-M-Y triplets to test the forward model, as this is the most commonly assumed model to explain genetic effects on both chromatin accessibility and gene expression [7]. The causal forward model was only tested when the ca/eQTL variant (FDR < 5%) was within the chromatin-accessible region, because such a variant in a gene regulatory region is likely to disrupt TF binding and then affect gene expression (Fig. 1b, Model 1 upper diagram). We found that in progenitors and neurons, 681 and 204 variants within 289 and 64 chromatin-accessible regions + / − 1 Mb from the transcription start site (TSS) of 332 and 83 genes were significantly associated with both chromatin accessibility and gene expression (Additional file 1: Fig. S1a).

In concordance with the previous observation of the sharing of directionality of genetic effect between caQTLs and eQTLs [13, 45], we detected that 85% and 80% of X-M-Y triplets showed allelic effects in the same direction on both chromatin and gene expression progenitor and neurons, respectively (Additional file 1: Fig. S1b). To evaluate causal forward relationships of genetic mediation of gene expression through chromatin accessibility, we applied a Bayesian mediation approach *bmediatR* [22] where X is a single variant within the chromatin accessibility peak, M is chromatin accessibility, and Y is the gene expression.

**Fig. 2:**
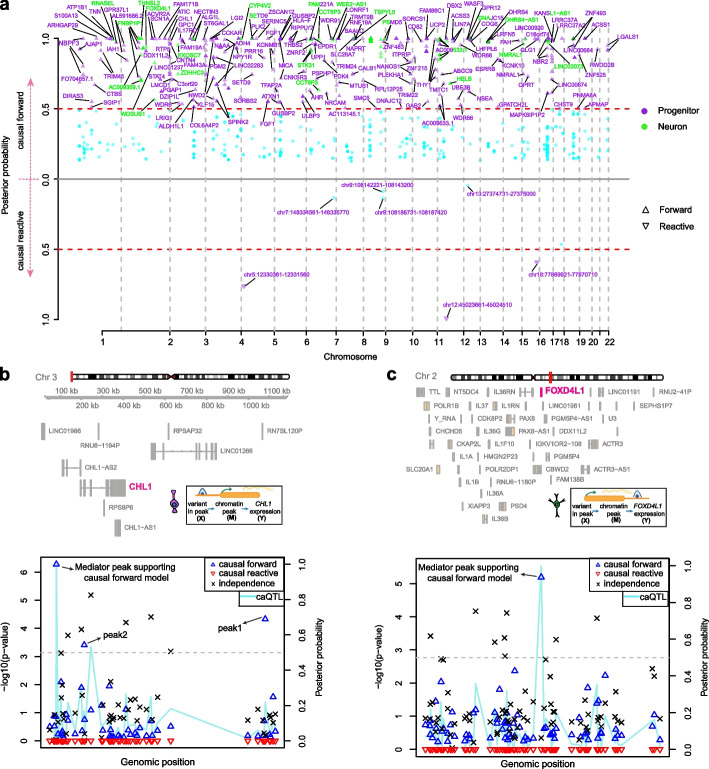
Cell-type-specific gene regulation mediated by chromatin accessibility. **a** Manhattan plot showing the *bmediatR* results for the causal forward model (upper), and for the causal reactive model (lower) for each cell type (purple for progenitor, green for neuron, purple + green for genes detected in both cell types). Gene symbols and chromatin-accessible regions are shown. **b** Mediation scan plot overlaid with caQTL data for the causal forward model of epigenetic regulation of *CHL1* gene expression in progenitors indicated by lines in different colors with corresponding caQTL *p*-value on the left y-axis, and the *bmediatR* posterior probability of each possible model is shown with different shapes on the right y-axis. The locations of peak harboring the variant, peak1 and peak2 supporting the causal forward model are highlighted. Dashed line indicates posterior probability = 0.5. **c** Mediation scan plot overlaid with caQTL data for the causal forward model of epigenetic regulation of *FOXD4L1* gene expression in neurons. Line and shapes are assigned as part b. Dashed line indicates posterior probability = 0.5

We detected 168 and 38 genes associated with 364 and 87 variants in progenitors and neurons supported by the causal forward model (posterior probability > 0.50; Fig. 2a, Additional file 2: Table S1). As an example where the Bayesian approach supported the causal forward model, an eQTL-caQTL colocalization in progenitors showed that variation of *CHL1* gene expression was found to be mediated through a chromatin accessibility peak (chr3:74,521–75,730) (Fig. 2b, posterior probability causal forward complete/causal forward partial: 0.40/0.59). The same progenitor eSNP and caSNP (rs9867864) within a chromatin accessibility peak located 121 kb upstream of the gene TSS was found to influence *CHL1* expression through altering chromatin accessibility, and showed allele-specific chromatin accessibility (ASCA) (FDR for ASCA = 1.27 × 10^−5^). The SNP did not survive our threshold for testing causal models in neurons, because rs9867864 was significantly associated with chromatin accessibility but not with gene expression, indicating that there were cell-type-specific impacts of this variant on gene expression (ca/eQTL nominal *p*-values in neurons = 1 × 10^−9^/0.019, Additional file 1: Fig. S2a). We also performed a mediation scan analysis substituting different chromatin-accessible regions (M) within + / − 1 MB of the TSS of the gene to test for specificity of the mediation effect (posterior probabilities for substituted regions M are shown in Fig. 2b). This analysis showed that the chromatin accessibility peak containing the variant had the highest posterior probability for forward mediation of the genetic effect on *CHL1* expression (Fig. 2b). Though 94% of peaks tested did not show evidence for mediating the effect, we detected two more chromatin accessibility peaks also surviving the threshold we used to determine causal forward models, although our caQTL analysis was not sensitive enough to detect the significant impact of rs9867864 on these chromatin-accessible regions (causal forward complete/causal forward partial for peak1 = 4.8 × 10^−7^/0.69; for peak2 = 1.1 × 10^−6^/0.54, nominal caQTL *p*-values for peak1 = 0.042, peak2 = 0.085, Fig. 2b). Both peak1 and peak2 were significantly associated with *CHL1* expression after controlling for the center peak and technical covariates (Additional file 1: Fig. S2b). This observation suggests that *CHL1* expression may be mediated by the peak harboring the variant along with peak1 and peak2. There have been case studies, though no definitive associations, suggesting that heterozygous deletion of *CHL1* showed language and cognitive developmental delay [54, 55]. Here, we propose a progenitor-specific causal regulatory mechanism for differences in *CHL1* expression. If the association with CHL1 and cognitive delay is confirmed, this finding may have therapeutic potential in that *CHL1* expression levels could be manipulated through targeting epigenetic engineering tools in progenitors to this enhancer region.

Another cell-type-specific mediation supporting the causal forward model was observed in neurons at the *FOXD4L1* gene locus. The variant rs141063413, within a chromatin-accessible region (chr2:113503031–113503850) located 2 kb downstream of the gene was significantly associated with *FOXD4L1* expression and chromatin accessibility in neurons (Fig. 2c). In progenitors, the same variant was associated with chromatin accessibility but not gene expression (ca/eQTL nominal *p*-values in progenitors = 6.9 × 10^−10^/0.12, Additional file 1: Fig. S2c). The chromatin-accessible region including the variant rs141063413 mediated *FOXD4L1* expression in neurons (causal forward complete/causal forward partial: 0.024/0.914, Fig. 2c). FOXD4L1 was shown to be required for embryonic neurogenesis in xenopus [56]. These results again suggest a cell type and a regulatory element that may be useful in modulating the expression of a given gene.

**Fig. 3:**
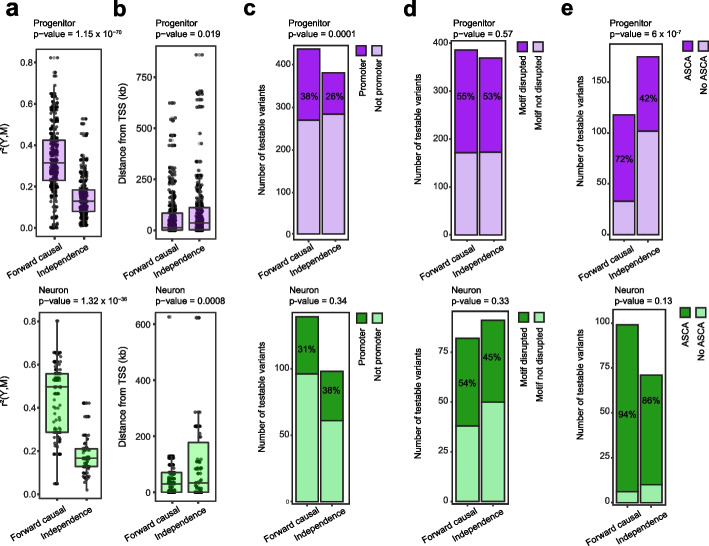
Biological and technical features predicting causality. **a** Percent variance in gene expression explained by chromatin accessibility (*r*^2^*(Y,M)*) in X-M-Y triplets supporting causal forward model versus independence in progenitors (upper, purple) and neurons (lower, green). Unpaired *t*-test *p*-value is shown. **b** Absolute distance of variants relative to TSS of the genes for X-M-Y triplets supporting causal forward model versus independence in progenitors (upper, purple) and neurons (lower, green). Unpaired* t*-test *p*-value is shown. **c** Distribution of variants located within gene promoter (within + / − 2 kb window from gene TSS) within X-M-Y triplets supporting causal forward model versus independence in progenitors (upper, purple) and neurons (lower, green). Chi-square test *p*-value is shown. **d** Number of testable variants detected to disrupt TF binding motifs via *motifbreakR* in X-M-Y triplets supporting causal forward model versus independence in progenitors (upper, purple) and neurons (lower, green). Chi-square test *p*-value is shown. **e** Number of testable variants (either variant itself or LD proxy) showed allele-specific chromatin accessibility (ASCA) in X-M-Y triplets supporting causal forward model versus independence in progenitors (upper, purple) and neurons (lower, green). Chi-square test *p*-value is shown

Next, we assessed the features that can be predictive of causal versus independent relationships between chromatin accessibility and gene expression regulated by the same locus. We found that as the relationship between chromatin accessibility and gene expression (percent variance in gene expression explained by chromatin accessibility termed *r*^*2*^*(Y,M)*) is stronger, causal forward models were more strongly supported in both cell types (Fig. 3a). We also observed that e/caQTLs supporting causal forward models were significantly closer to the gene TSS in both cell-types and enriched more within promoters in progenitors (Fig. 3b, c). However, we did not detect enrichment of testable variants disrupting TF binding motifs [57] within the causal forward model compared to the independence model (Fig. 3d). The variants supported by ASCA were also more enriched within the causal forward model compared to the independence model in progenitors, but not in neurons (Fig. 3e). This suggests that the amount of variance in gene expression explained by chromatin accessibility, genomic location relative to gene TSS and ASCA can be predictive features for causality. However, TF motif disruption is not a reliable feature for the assumption of causality. It is important to note that low power to detect ASCA based on allele frequency, incomplete annotation of motifs, and incomplete knowledge of how genetic variation disrupts TF binding, all prevent a comprehensive evaluation of these features.

A comparison of triplets supporting the causal forward model using the Bayesian approach (bmediatR) with regression-based mediation analysis [44] showed that 90.7% and 23.7% of the triplets in progenitors and neurons overlapped with the triplets supporting causal forward models determined by a complementary regression-based approach (Additional file 1: Fig. S3a). We hypothesized that lower statistical power may at least partially explain the lower agreement between the two approaches in neurons. To this end, we down-sampled progenitors to be equal in sample size with neurons, and as expected we found a decreased overlap between Bayesian versus regression-based mediation approaches (Additional file 1: Fig. S3b, 47.5% overlap). This suggests that there may be general agreement between the two approaches for studies with a sufficient sample size.

Given that sample size may influence causality inferences, we sought to assess replication in another brain tissue dataset where multiple gene regulatory modalities and genotypes were acquired on the same individuals (ROSMAP/xQTL) [43]. To assess replication of our cell-type-specific ca/eQTL data in relevant datasets, we computed *π*_1_ statistics [58] within adult brain methylation QTL and eQTL data from the previous study [43] for significant primary variant-chromatin accessibility and variant-eGene pairs. We found that the fraction of progenitor and neuron primary SNP-overlapping epigenetic regulatory region pairs that are non-null associations in adult brain ROSMAP/xQTL methylation QTL data (*π*_1_) was 56.7% and 24.1% when subsetting to SNP-epigenetic regulatory region pairs that were detectable in both datasets (Additional file 1: Fig. S4a). Also, we observed that the fraction of progenitor and neuron primary eSNP-eGene pairs that are non-null associations in ROSMAP/xQTL eQTL data (*π*_1_) was 77.1% and 86.6% when subsetting to SNP-Gene pairs detectable in both datasets (Additional file 1: Fig. S4a). Estimates for sharing of ca/mQTLs and eQTLs across datasets were well above randomly sampled SNPs in ROSMAP/xQTL data, which showed nonsignificant associations in our cell-type-specific data, demonstrating clear evidence for replication of the QTL findings.

We also compared the mediation analysis results between our study and the ROSMAP/xQTL study [43]. We detected 7 and 1 SNP-chromatin accessibility peak-eGene triplets in progenitor and neuron-supported mediation in a causal forward direction in both our analysis and xQTL study (Additional file 1: Fig. S4b, Additional file 2: Table S1). As a specific example, we observed a causal regulatory region detected in progenitor, neuron, and ROSMAP/xQTL data at the *DNAJC15* gene locus (Additional file 1: Fig. S4c). We found that variant rs17553284 within a chromatin accessibility region at gene promoter was a caQTL and an eQTL in both progenitor and neuron data, and a mQTL and an eQTL in ROSMAP/xQTL data. While the C allele of the variant increased *DNAJC15* expression in progenitor, neuron, and ROSMAP/xQTL data, it decreased DNA methylation and increased chromatin accessibility in progenitor and neuron data consistent with the opposite regulatory direction of DNA methylation and chromatin accessibility (Additional file 1: Fig. S4d). Importantly, the chromatin-accessible region detected in our cell-type-specific data mediated *DNAJC15* expression in both progenitor and neurons, also DNA methylation site detected in ROSMAP/xQTL data mediated *DNAJC15* expression (bmediatR posterior probability for causal forward: progenitor = 0.99, neuron = 0.99 and causal inference test (CIT) [59] *p*-value in xQTL for causal forward = 5.9 × 10^−7^ and for causal reactive = 0.001, Additional file 1: Fig. S4e). Despite differences in sample age, heterogeneity, and method used for mediation analysis, observing the replication between two datasets suggested our approach is robust.

### Accuracy of Bayesian mediation approach in classifying forward versus reactive models given differences in measurement error between phenotypes

Given that differences in measurement error between candidate mediators and outcomes may give rise to false positive causal relationships [20, 23], we quantified measurement error using the intraclass correlation coefficient (ICC) [60] across technical replicates from the same donor line thawed multiple times. We observed that ICC for ATAC-seq measured peaks (*N*_donors_with_replicates_ = 11 in progenitors, *N*_donors_with_replicates_ = 5 in neurons) were on average lower than RNA-seq measured genes (*N*_donors_with_replicates_ = 13 in progenitors, *N*_donors_with_replicates_ = 9 in neurons) (Additional file 1: Fig. S5a), indicating higher measurement error in ATAC-seq data. We simulated the impact of measurement error on causal models to find the ICC at which true causal forward or reactive models flip to being falsely called reactive or forward models. Since we observed that the ICC flipping threshold was also dependent on the magnitude of the effects of X on M and Y on M, we calculated an ICC threshold by varying the magnitude of effects for X-M-Y triplets. For example, in a simulated causal forward model where variant X explains 30% of the variation in chromatin accessibility (M) and the variant X also explains 10% of the variation in gene expression (Y), we find that ICC values of chromatin accessibility below 0.3 lead to incorrect model flipping (Additional file 1: Fig. S5b-c; see the “Methods” section). We filtered out the triplets supporting causality with ICC values lower than per triple threshold of ICC at which model flipping occurred for all future analyses (Additional file 1: Fig. S5b, see the “Methods” section).

We then subset the ca/eQTLs to biologically relevant X-M-Y triplets to test the reactive models. The causal reactive model was only tested when the ca/eQTL variant altered the expression of a TF and the chromatin accessibility of a region harboring a motif for that TF, as this is a likely mechanism for a causal reactive model (Fig. 1b, Model 1 lower diagram). We detected 3 variants cis-regulating 3 genes encoding TFs and also associated with chromatin accessibility within 8 regions that contained the binding motif of the TF in progenitors (Fig. 2a). We did not detect any significant ca/eQTLs matching our causal reactive model criteria in neurons (at 5% FDR for trans-caQTLs).

We would expect that in true reactive models, where a variant’s effect on chromatin accessibility is fully mediated through gene expression, no allelic imbalance in chromatin accessibility would be observed by X or an LD proxy of X, because the effect is not *cis* with respect to the chromatin peak, but *trans*. Hence, we excluded variants within chromatin-accessible regions to evaluate causal reactive models. We investigated a few cases where the reactive model had a higher posterior probability than the forward model. As an example, the variant rs2731040 located 1 kb upstream, not within, a chromatin-accessible region (chr12:45023661–45024510) harboring several DBX2 TF binding motif sites was significantly associated with both *DBX2* expression and chromatin accessibility (Additional file 1: Fig. S6a-b). This example satisfied our previously defined biologically motivated criteria to test whether the data support a causal reactive model. The same chromatin-accessible region additionally harbored binding motifs of 15 different TFs, the expression of each of which was cis-regulated. When we tested if the variants cis-regulating expression of these TFs were also significantly associated with chromatin accessibility at this peak, we did not detect any significant trans-caQTL loci other than rs2731040, the variant that was also associated with *DBX2* TF expression (Additional file 1: Fig. S6c). Data from this X-M-Y triplet best fit the causal reactive model (posterior probability for causal forward/causal reactive: 0.0095/0.99). This observation initially suggested a potential autoregulatory mechanism for a reactive model whereby expression of *DBX2* regulated by the rs2731040 variant subsequently led to a change in chromatin accessibility. However, we also found another variant, rs2731038, within the chromatin-accessible region that was in LD with rs2731040 (*r*^2^ = 0.66), and rs2731038 showed ASCA in our previous study (Additional file 1: Fig. S6d, the adjusted *p*-value for ASCA = 0.02) [13]. Given that ASCA indicates a forward regulatory mechanism for chromatin accessibility by the variant, we interpret the high posterior probability supporting the reactive model as a false positive result. This observation shows that causal reactive models must be carefully evaluated to ensure that they are not tagging allele-specific effects, which are indicative of causal forward models. Through careful follow-up of X-M-Y triplets, we demonstrate that false positive reactive models may persist despite stringent criteria and posterior probability thresholding.

### *Cell-type-specific mediation of eQTLs *via* trans-regulation*

**Fig. 4:**
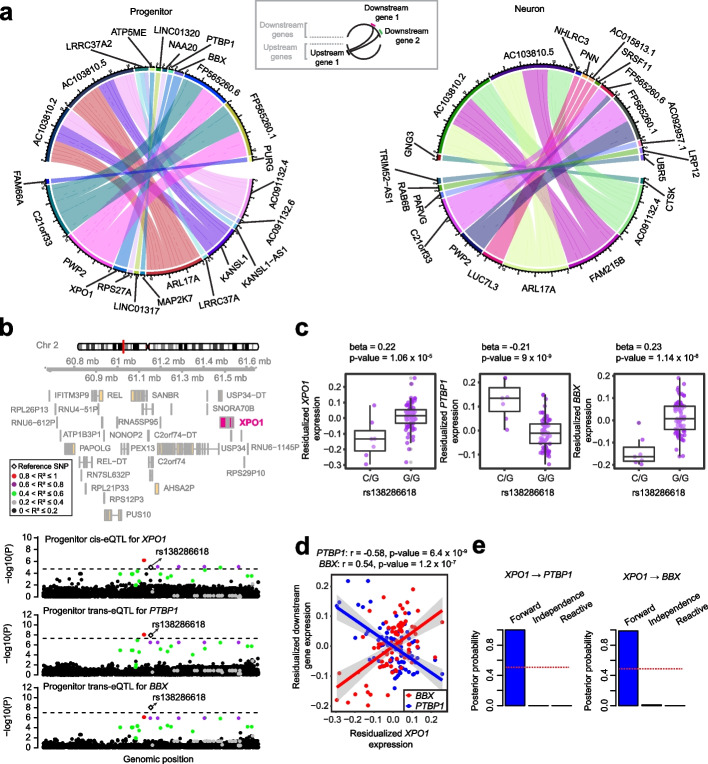
Cell-type-specific gene expression mediated by trans-regulation. **a** Circle plots illustrating genes mediated by trans-regulation in progenitors (left) and in neurons (right). Upstream genes (external circle color matches line color) are shown interacting with downstream genes (external circle color does not match line color). **b** Genomic tracks illustrating the association of variants cis-regulating *XPO1* upstream gene with the expression *XPO1*, and downstream genes *PTBP1* and *BBX*. Data points were colored based on the pairwise LD *r*^2^ with the rs138286618 (reference SNP). Dashed lines indicate the *p*-value threshold for significance in cis-eQTL or trans-eQTL analysis. **c** Boxplots showing the relationship between expression of genes residualized by the covariates and rs138286618 variant. **d** Correlation between *XPO1* vs *PTBP1* genes and *XPO1* vs *BBX* genes. Residualized expression by covariates are shown for all genes. **e** *bmediatR* posterior probability for causal forward, independence versus causal reactive models for the regulation of *PTBP1* and *BBX* genes by *XPO1*. Posterior probabilities of causal reactive were set to zero by *bmediatR* since reactive model priors were not evaluated for mediation via trans-regulation

Next, we investigated genes that were mediated via trans-regulation for each cell-type using the same Bayesian mediation approach. Because this analysis did not require both ca/eQTL data derived from the same donor, sample sizes were slightly increased as compared to the previous analyses (*N*_donor_ = 85 in progenitors and *N*_donor_ = 74 in neurons) (Fig. 1b, Model 2). Within each cell-type, SNPs with significant association to a proximal gene (cis-eSNPs, primary SNPs per gene as well as pruned SNPs, see Methods) were also tested for association with every other gene (trans-eQTL, see the “Methods” section). At a 10% FDR significance threshold, we detected 35 and 30 variants cis-regulating 23 and 17 upstream genes and also associated with 22 and 21 downstream genes in progenitor and neurons, respectively. We assessed whether cell-type-specific trans-eQTL results were replicated in fetal bulk brain data (*N*_donor_ = 235) [14, 15]. The fraction of significant progenitor and neuron eSNP-trans (downstream) gene pairs that are non-null associations in fetal bulk trans-eQTL data (*π*_1_) was estimated to be 95.5% and 74.2% when subsetting to SNP-Gene pairs detectable in both datasets, providing strong evidence for replication. After performing mediation analysis, we discovered 11 and 12 downstream genes that were mediated via trans-regulation by 13 and 10 cis-regulated upstream genes in progenitors/neurons, respectively (Fig. 4a; posterior probability for causal forward > 0.5, Additional file 3: Table S2).

As an example, *XPO1* on chromosome 2, was cis-regulated by a progenitor-specific eSNP (rs138286618), and the same variant was associated with the expression of genes *PTBP1* on chromosome 19 and *BBX* on chromosome 3 (Fig. 4b and c). *PTBP1* and *XPO1* genes were positively correlated; whereas *BBX* and *XPO1* genes were negatively correlated (Fig. 4d). The mediation analysis showed that the genetic effects of the variant on *PTBP1* and *BBX* were mediated via *XPO1* gene expression (posterior probabilities supporting causal forward = 0.99 and 0.99 for both *PTBP1* and *BBX*, Fig. 4e). XPO1, which is a nuclear export protein [61], induced apoptosis of cortical neural progenitors in mice [62] and intronic mutations within XPO1 were associated with autism [62, 63]. PTBP1 protein is an RNA-binding protein, which was upregulated in glioblastoma [64] and had a role in the regulation of neuronal differentiation [65]. BBX protein is a transcription factor which is highly expressed in progenitor cells of developing neocortex and found as a downstream target of NFIX transcription factor regulating neural development [66]. *XPO1* may therefore represent an important master regulator of disease-associated genes in progenitors during neural development.

### *Proposing regulatory mechanisms of brain-related GWAS loci *via* genetically mediated gene expression*

**Fig. 5:**
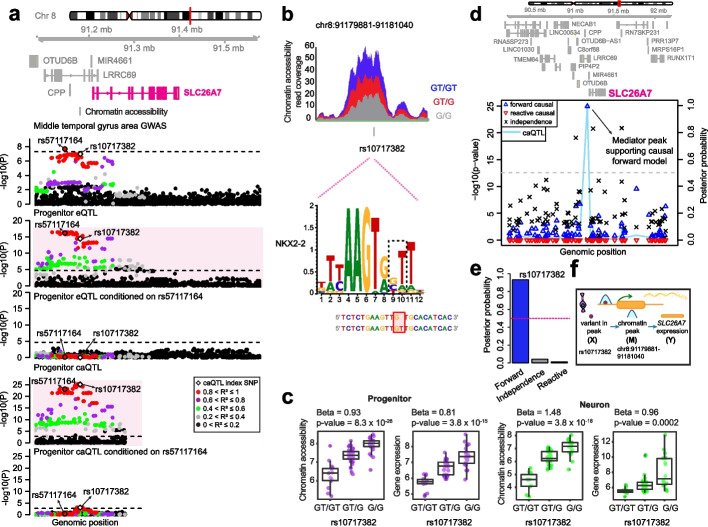
Colocalization of shared ca/eQTLs at *SLC26A7* locus with middle temporal gyrus area GWAS. **a** Genomic tracks illustrating regional association of variants with *SLC26A7* gene expression, chromatin accessibility at its promoter, and middle temporal gyrus area GWAS. Colocalization of middle temporal gyrus area GWAS index SNP with shared ca/eQTL SNP was detected via conditional analysis. Data points were colored based on the pairwise LD *r*^2^ with the caQTL index variant rs10717382 within the chromatin-accessible region. The dashed lines correspond to *p*-value thresholds for significance for each dataset. **b** Coverage plot illustrating ATAC-seq reads within the chromatin-accessible region per genotype. Genomic position of the variant rs10717382 was shown along with the NKX2-2 TF motif disrupted by rs10717382. **c** Phenotype (chromatin accessibility and *SLC26A7* expression) versus genotype boxplots per cell type. **d** Mediation scan plot overlaid with caQTL data for the causal forward model of epigenetic regulation of *SLC26A7* expression in progenitors indicated by lines in different colors with corresponding *p*-value on the left y-axis, and the posterior probability of each possible model shown with different shapes on the right y-axis. The location of the peak supporting the causal forward model was highlighted. **e** *bmediatR* posterior probability for causal forward, independence versus causal reactive models for the regulation of *SLC26A7* by chromatin accessibility. Posterior probabilities of causal reactive were set to zero by *bmediatR* since reactive model priors were not evaluated for mediation via chromatin accessibility. **f** The cartoon illustrating a causal forward model for the regulation of *SLC26A7* by chromatin accessibility

We further leveraged cell-type-specific causal pathways to interpret the function of GWAS loci associated with brain-relevant traits (Additional file 4: Table S3). As a specific example, we observed that an indel variant (rs10717382) was significantly associated with chromatin accessibility at a peak (chr8:91179881–91181040) upstream of the *SLC26A7* gene as well as its expression in progenitors. The variant rs10717382 was also co-localized with an index SNP (rs57117164) associated with inter-individual differences in the surface area of a specific cortical region, the Middle Temporal Gyrus [4] for progenitor eQTLs, also the significance of caQTL dramatically reduced upon conditioning on rs57117164 (*p*-value was changed from 8.3 × 10^−26^ to 0.0009) (Fig. 5a). Importantly, we detected that deletion of the T allele decreased the binding affinity of the NKX2-2 transcription factor based on in silico analysis (Fig. 5b) [57]. NKX2-2 was previously found to function as a transcriptional repressor [67–69], and consistent with this, we observed that the deletion of the T allele was also associated with increased chromatin accessibility and gene expression in progenitor cells, but did not survive correction for multiple comparisons for association with expression in neurons (Fig. 5c). The deletion of T allele was also associated with increased Middle Temporal Gyrus area. Mediation analysis showed that this variant impacts *SLC26A7* gene expression through chromatin accessibility (Fig. 5d–e, posterior causal forward complete/causal forward partial: 0.028/0.966). *SLC26A7* encodes an anion transporter and mutations in the SLC26A7 protein were found in individuals with congenital hypothyroidism, though the role of this gene in brain structure is unclear [70–72]. Overall, here we proposed a cell-type-specific genetic causal path where regulation of the *SLC26A7* gene impacts brain structure, possibly through thyroid metabolism, that can be experimentally validated in future studies.

## Discussion

Here, integrating multiple QTLs, we identified cell-type-specific causal gene regulatory mechanisms in a model system of the developing human brain. Our approach demonstrated several important features of gene regulation: (1) testing causality for shared QTLs between chromatin accessibility and gene expression and between cis- and trans-regulated genes provided insights into epigenomic and transcriptomic features of gene regulatory networks by linking variant to peak to gene and variant to gene to gene; (2) utilization of a cell-type-specific system allowed us to observe context-dependent causal networks that may have been masked by tissue heterogeneity in previous causal inference efforts on bulk tissue from the human brain; (3) applying a Bayesian strategy rather than traditional regression-based mediation, we could evaluate models of genetic effects on gene expression via chromatin accessibility in terms of the posterior probability of fully, partially, or independent mediation.

Different molecular assays require unique experimental procedures resulting in different levels of technical noise across molecular phenotypes, which may bias estimates of mediated effects [23, 73]. Importantly, based on simulations, we demonstrated that reliable measurements are required to make accurate conclusions about causal mechanisms after data integration. We attempted to eliminate false positive causal relationships by implementing an algorithm that detects a threshold ICC value. We observed that forward models can be identified as reactive at low ICC values; whereas reactive to forward model flipping is very unlikely to occur. Several adjustments including larger sample sizes, more donors with replicates, and a higher read coverage for sequencing data might reduce the measurement error. Moreover, despite our attempt to avoid potential false positive reactive models via this strategy, we were not able to identify any biologically meaningful examples of reactive candidates. This observation may be interpreted in light of the fact that (1) observation of a reactive model, chromatin accessibility mediated by gene expression, is an unlikely biological mechanism in this dataset, and (2) small sample size and measurement error may obscure distinguishing partial forward and partial reactive, as observed by the authors of the *bmediatR* method in simulation analyses [22]. We expect that increasing the statistical power of QTLs using a larger number of samples will be helpful to determine how often, if at all, reactive regulatory models exist.

We explored a variant co-localized with an index SNP for the middle temporal gyrus area upstream of the *SLC26A7* gene, which alters chromatin accessibility at the region leading to changes in gene expression. This observation directed us to propose a comprehensive mechanism to interpret how a GWAS locus alters complex brain structure*.* Despite the low number of QTLs that colocalized with any brain-relevant GWAS loci, we detected cis-regulatory elements mediating the expression of genes whose haploinsufficiency is associated with rare neurodevelopmental conditions. For instance, we found that a genetically altered chromatin-accessible region upstream of the *CHL1* gene mediated its expression. Heterozygous loss of the *CHL1* gene has been observed in individuals with cognitive delays [54, 55], though a definitive statistical association has not yet been conducted. Upon validation by cellular assays, this regulatory region could be an appealing candidate upstream enhancer to modulate endogenous gene expression for CRISPR-based targeted therapeutic approaches [74–77]. In addition to epigenetic regulations, we identified a downstream mediation of the *PTBP1* and *BBX* genes by the *XPO1* gene. Using the genetic variation within this region as an instrumental variable serving as a natural perturbation [78], we identified a causal relationship between two genes that would not be possible to infer by merely considering their co-expression. All of these three genes have been involved in neocortical differentiation, and exploring their regulatory mechanisms can help to find novel neurodevelopmental abnormality-relevant intracellular pathways.

Although our sample size was comparable with other cell-based QTL studies [79–82], increasing the number of donors will likely lead to a higher number of shared QTLs enabling a more systematic and higher-powered mediation analysis. Also, since the detection of some QTLs is dependent on the presence of stimuli, this may prevent simultaneous observation of changes in chromatin accessibility and gene expression [83, 84]. To this end, further studies using more context-dependent conditions can be complementary to our findings with an objective to examine causal networks that connect risk variants associated with brain-relevant traits to cellular function.

## Conclusion

In this study, we identified epigenetic and trans-regulated causal pathways for the underlying mechanism of gene expression, employing a cell-type specific system representing an important period of human cortical development. Leveraging these causal networks with brain-relevant GWASs, we proposed potential molecular functions for trait-associated variants, which represent novel candidates for mechanistic studies aiming to understand inter-individual differences in neurodevelopmental traits.

## Methods

### Establishment of primary human neural progenitor cells (phNPCs)

We generated phNPCs and differentiated them into neurons following the same cell culture procedure as described in our previous work [13, 14, 85].

### Generation of cell-type-specific ATAC-seq and RNA-seq data

As previously described [4], we prepared ATAC-seq libraries and sequenced some of the libraries by using Illumina HiSeq2500 and some of them via MiSeq platforms with 50 bp paired-end sequencing with an average depth of 25.5 million read pairs. We aligned them to the human genome (GRCh38/hg38) by reducing mapping bias via the WASP method after quality control as described previously [4]. We generated RNA-seq libraries, performed sequencing with NovaSeq S2 flow cell using 150 bp paired-end sequencing with an average read depth of 99.8 million read pairs, and also mapped them to the human genome (GRCh38/hg38) after quality control as previously described [18].

### Genotype processing and imputation

We performed genotyping of some of the DNA libraries via Illumina HumanOmni2.5 and some of them via HumanOmni2.5Exome platforms. If variants showed variant missing genotype rate > 5% (–geno 0.05), deviations from Hardy–Weinberg equilibrium at *p* < 1 × 10^−6^ (–hwe 10^−6^), and minor allele frequency < 1% (–maf 0.01), we filtered them out. We also excluded samples if they had missing genotype rate > 10% (–mind 0.10) as described previously [13, 14]. For imputation, we used 1000 Genomes Project Phase 3 reference panel for multiple ancestries [86] using Minimac4 software [87] by retaining variants with missing genotype rate lower than 0.05, Hardy–Weinberg equilibrium *p*-value greater than 1 × 10^−6^, minor allele frequency (MAF) bigger than 1% and imputation *R*^2^ greater than 0.3 as described previously [13, 14].

### Intraclass correlation within chromatin accessibility and gene expression data

To quantify cell culture-induced noise, we cultured 11 and 5 donors in progenitors and neurons to prepare ATAC-seq libraries and 11 and 9 donors in progenitors and neurons to prepare RNA-seq libraries multiple times during the course of the experiment. We calculated the intraclass correlation coefficient (ICC) of gene expression and chromatin accessibility between libraries from the same donors. For neurons, we used gene expression values after batch correction with the limma R package [88] for the sorter type, as described previously [13, 14]. We performed an unpaired two-sided t-test for statistical assessment of the mean difference between these two categories (Additional file 1: Fig. S5a).

### Cell-type-specific eQTL and caQTL mapping to test causal forward model

To find the candidate SNP-chromatin accessibility-gene candidates to be tested for causal forward model or independence, we tested the association of 171,256 and 161,716 variants located within chromatin-accessible regions and within + / − 1 MB of gene transcription sites both with 55,473 and 54,044 chromatin accessibility regions via caQTL analysis, and with 22,710 and 22,696 genes via eQTL analysis in progenitor and neurons, respectively. To perform QTL analyses, we utilized a linear mixed effects regression model via EMMAX software [89] in which we controlled for population stratification (ancestry) and cryptic relatedness. To control for population structure, we computed the first 10 MDS components of ancestry for all the individuals with genotype information available via PLINK software with (parameters: –cluster –mds-plot 10). To control cryptic relatedness, we generated kinship matrix from non-imputed genotype data via emmax-kin -v -h -d algorithm in EMMAX software, in which we excluded the genetic variants on the same chromosome with the tested variants in QTL analysis [90]. We controlled for unknown technical confounders by using the same number of PCs of global chromatin accessibility and gene expression models as previously established for caQTLs [13] and eQTLs [14] for each cell type. In these studies, the number of PCs was determined as the number maximizing caQTL discovery for caQTL analysis at 5% FDR and as the number maximizing eGene discovery at 5% FDR for eQTL analysis by sequentially adding PCs into the covariate matrix. The models were specified as follows, where for each model a random effect was included with covariance equal to the kinship matrix K multiplied by the genetic variance:

$$\mathrm{Progenitor}\;\mathrm{caQTL}:\mathrm{chromatin}\;\mathrm{accessibility}\sim\mathrm{SNP}+10\;\mathrm{MDS}\;\mathrm{of}\;\mathrm{global}\;\mathrm{genotype}+8\;\mathrm{PCs}\;\mathrm{of}\;\mathrm{global}\;\mathrm{chromatin}\;\mathrm{accessibility}$$

$$\mathrm{Neuron}\;\mathrm{caQTL}:\mathrm{chromatin}\;\mathrm{accessibility}\sim\mathrm{SNP}+10\;\mathrm{MDS}\;\mathrm{of}\;\mathrm{global}\;\mathrm{genotype}+\mathrm{FACS}\;\mathrm{sorter}+7\;\mathrm{PCs}\;\mathrm{of}\;\mathrm{global}\;\mathrm{chromatin}\;\mathrm{accessibility}$$

$$\mathrm{Progenitor}\;\mathrm{eQTL}\;:\;\mathrm{expression}\sim\mathrm{SNP}+10\;\mathrm{MDS}\;\mathrm{of}\;\mathrm{global}\;\mathrm{genotype}+10\;\mathrm{PCs}\;\mathrm{of}\;\mathrm{global}\;\mathrm{gene}\;\mathrm{expression}$$

$$\mathrm{Neuron}\;\mathrm{eQTL}\;:\;\mathrm{expression}\sim\mathrm{SNP}+10\;\mathrm{MDS}\;\mathrm{of}\;\mathrm{global}\;\mathrm{genotype}+\mathrm{FACS}\;\mathrm{sorter}+12\;\mathrm{PCs}\;\mathrm{of}\;\mathrm{global}\;\mathrm{gene}\;\mathrm{expression}$$

We applied the same hierarchical multiple testing correction strategy as previously by computing a global eigenMT-FDR *p*-value after local adjustment per eGene [14]. To perform the eigenMT-FDR procedure [91], briefly, (1) nominal *p*-values of all cis variants per gene were locally adjusted via the eigenMT method [92]. (2) We performed multiple testing correction by using these locally adjusted* p*-values from the top SNPs per gene, which resulted in globally adjusted *p*-values. (3) Genes with globally adjusted* p*-value lower than 0.05 were defined as eGenes. For caQTLs, we adjusted association *p*-values via only the Benjamini–Hochberg method given that there were a few chromatin-accessible regions with multiple variants and retained the associations with lower than 5% FDR.

### Cell-type specific eQTL and caQTL mapping to test causal reactive model

To find the candidate SNP-chromatin accessibility-gene triplets to be tested for causal reactive model against independence, we applied the following strategy: (1) we detected (i) eGenes (53 eGenes in progenitors and 12 eGenes in neurons) that encode transcription factors with known motifs [93] by calculating eigenMT-FDR threshold *p*-value by using only eGenes encoding TFs, and (ii) variants cis-regulating these eGenes at this significant threshold; (2) we searched chromatin-accessible regions throughout the genome that harbor 80% matching sequence of the binding motifs [93] of these TF eGenes via TFBSTools [94]; and (3) we performed trans-caQTL analysis by using variants from step 1 and chromatin accessibility at the regions from step 2 with the same caQTL models described for the causal forward model. We defined significant trans-caQTLs at 5% FDR.

### Cell-type specific trans-eQTL mapping

To perform trans-eQTL analysis, we first selected a list of variants with significant evidence of cis-regulation for at least one gene in our previous analysis (*N*_donor_ = 85 in progenitors and *N*_donor_ = 74 in neurons) [14]. We further included variants with minor allele frequency greater than 0.025 and included primary SNPs associated with cis genes and pruned variants via PLINK v.1.90b3 software [95] (parameters –indep-pairwise 50 5 0.5). We excluded cis- or trans-pseudogenes, and any upstream–downstream gene pairs if they had a cross-mappability value [96] greater than 5 at log2 scale. For trans-eQTL analysis, we included variant-gene pairs if the distance between the variant and TSS of the gene was larger than 1 MB on the same chromosomes or if the variant was on a different chromosome than the gene. We tested associations of the filtered variants with all the filtered genes in the genome that were expressed in our dataset. To conduct trans-eQTL analysis, we utilized EMMAX software [89] controlling for population structure and unknown technical confounders by adding the number of PCs of global expression maximizing eGene discovery in our previous cis-eQTL analysis [14]. The following models were used for trans-eQTL analysis per cell type, where for each model a random effect was included with covariance equal to the kinship matrix K multiplied by the genetic variance:

$$\mathrm{Progenitor}\;\mathrm{eQTL}\;:\;\mathrm{expression}\sim\mathrm{distal}\;\mathrm{SNP}+10\;\mathrm{MDS}\;\mathrm{of}\;\mathrm{global}\;\mathrm{genotype}+10\;\mathrm{PCs}\;\mathrm{of}\;\mathrm{global}\;\mathrm{gene}\;\mathrm{expression}$$

$$\mathrm{Neuron}\;\mathrm{eQTL}\;:\;\mathrm{expression}\sim\mathrm{distal}\;\mathrm{SNP}+10\;\mathrm{MDS}\;\mathrm{of}\;\mathrm{global}\;\mathrm{genotype}+\mathrm{FACS}\;\mathrm{sorter}+12\;PCs\;\mathrm{of}\;\mathrm{global}\;\mathrm{gene}\;\mathrm{expression}$$

To detect significant trans-eQTLs, we performed multiple testing correction and defined the associations as significant at 10% FDR.

### Bayesian approach for mediation analysis

For mediation of gene expression through chromatin accessibility: for each X-M-Y triplet where X is a genetic variant (encoded as − 1,0,1) within a chromatin accessibility region that is significantly associated with both chromatin accessibility and gene expression; M is the chromatin accessibility and Y is the gene expression, we ran mediation analysis applying bmediatR [22] using the covariates for caQTL and eQTL data corresponding to population structure and technical factors. We applied the default setting for hyperparameters representing the effect sizes to φ^2^ = (1, 1, 1) relationships between X and M, M and Y, and X and Y, respectively, assuming them to be equal a priori for each, since we observed that φ^2^ = (1, 1, 1) as one of the values maximizing the sum of marginal log likelihoods (Additional file 1: Fig. S1c). To test the causal forward model, we set the ln_prior_c parameter for model prior as “complete” by assuming that observation of the causal reactive model is not plausible. To evaluate the causal reactive model and simulations used to remove false positive causal relationships, it was set as “reactive” to include reactive model priors [22]. We used the non-informative default priors for the scaling parameters (κ, λ) = (0.001, 0.001) and fixed effect coefficients, both the intercept and covariates τ = (1000, 1000). This analysis calculated the posterior distribution of θ, which denotes the edges of the DAG relating X-M-Y) by multiplying a joint likelihood for Y and M with a prior distribution for θ as p(θ|y,m) α p(y,m|θ)p(θ). We defined the relationship as causal forward if the sum of the posterior probabilities of complete and partial mediation was higher than 0.5, and as reactive if the sum of the posterior probabilities of complete reactive and partial mediation models was higher than 0.5.

To limit potential false positive (FP) causal relationships that may result from imbalanced measurement error across M and Y, we simulated normally distributed random variables introducing variable error added to M (M’) and Y (Y’). We then detected false positive causal reactive and forward mediation, respectively, using percent of variance of M explained by X (PVE_A) and the percent of variance of Y explained by M on the odds scale (PVE_B) per X-M-Y triplet from the real data. We performed analysis separately for each scenario of model flipping: (1) from forward to reactive to detect FP reactives, and (2) from reactive to forward to detect FP forwards. After running mediation analysis, we fitted the posterior probabilities supporting forward and reactive models against ICC values changed upon application of the error term via local polynomial regression. Following the fitting, we attempted to define an ICC threshold at which the model flips from forward to reactive or from reactive to forward. To this end, we calculated the distance between the two curves corresponding to two models and defined the ICC value that minimized the distance if the slope of the wrong model is positive and the slope of the correct model is negative relative to ICC values bigger than this threshold (Additional file 1: Fig. S5c). We performed this simulation 10 times and averaged ICC threshold values per X-M-Y triplet whose corresponding PVE_A and PVE_B values were used during the simulation. We filtered out X-M-Y triplets with ICC for chromatin accessibility was lower than the threshold ICC value for the results supporting the reactive model, respectively. Additionally, we retained only X-M-Y triplets with positive ICC values for chromatin accessibility and gene expression or both models. We detected that model flipping from reactive to forward was only possible when both PVE_A and PVE_B values were bigger than 0.9 (data not shown). Since we did not have such large PVE values for X-M-Y triplets tested in our study, we did not need to apply this algorithm to limit potential false-positive forwards.

For mediation of gene expression through the expression of a distal gene, each X-M-Y triplet where X is a genetic variant that is significantly associated with the expression of both genes; M is the gene cis-regulated by X and Y is the gene trans-regulated by X, similarly, we applied bmediatR [22]. We used the covariates for eQTL data corresponding to population structure and technical factors with the same hyperparameters described for mediation through caQTL above. Given that the reactive model was not biologically interpretable for trans-regulation, we only considered the forward direction for causality, and we considered the relationship as causal forward if the sum of the posterior probabilities of complete and partial mediation were higher than 0.5.

### Regression-based approach for mediation analysis

We additionally applied a traditional mediation approach with regression analysis to detect the mediation of genetic effects on gene expression via chromatin accessibility. For each cell type, we established the linear models where Z_Y_ and Z_M_ are the covariate matrices used for eQTL and caQTL analyses, M_residualized_ is chromatin accessibility residualized by Z_M_ and Y_residualized_ is gene expression residualized by Z_Y_ and tested alternative hypothesis H_1_ below:

For causal forward model:

$${H}_{0} :Y \sim X + {Z}_{y}$$

$${H}_{1} :Y \sim X+ {Z}_{y}+{M}_{\mathrm{residualized}}$$

We performed multiple testing applying a mediation scan strategy as previously described [44]. For a causal forward model, per each gene–SNP pairs, we randomly selected chromatin accessibility regions from another chromosome to condition on. Then after permuting the mediator 1000 times for the sample index, we selected the maximum conditional *p*-value (the least significant) from each permutation and fit a generalized extreme value distribution (GEV). Finally, we calculated the FWER-controlled mediation *p*-values using the cumulative density function of the GEV and set a significance threshold of 0.05.

To evaluate a forward model with a high partial mediation probability for *CHL1* gene regulation (Additional file 1: Fig. S2c), we applied a similar strategy. We tested if there was any significant association between residualized *CHL1* expression by both technical covariates and chromatin accessibility at the peak harboring the variant (that was also residualized by technical covariates), and peak 1 and 2 chromatin accessibilities, separately (that were also residualized by technical covariates).

### Replication of ca/eQTL data and mediation analysis in the ROSMAP/xQTL data

To assess the replication of our ca/eQTL data in the ROSMAP/xQTL data [43], we initially liftovered the positions of methylation sites and variants from hg19 to hg38. Within this dataset, we detected methylation sites or CpG islands harboring the methylation sites (obtained from Illumina HumanMethylation450 manifest file) overlapped with chromatin accessibility peaks in our caQTL data and defined these as overlapping regulatory regions. We estimated the fraction of progenitor and neuron primary SNP-overlapping regulatory region pairs that are non-null associations in adult brain ROSMAP methylation QTL data by using the corresponding *p*-values to SNP-regulatory region pairs that were detectable in both datasets via *π*_1_ statistics [58]. For the comparison of our cell-type-specific eQTL data with adult brain ROSMAP eQTL data, we estimated the fraction of progenitor and neuron primary eSNP-eGene pairs that are non-null associations in ROSMAP eQTL data (*π*_1_) by using the corresponding *p*-values to SNP-Gene pairs detectable in both datasets. To demonstrate that the *π*_1_ value calculated via non-null associations was not by chance, we also randomly sampled an equal number of SNP-regulatory region pairs or SNP-gene pairs in ROSMAP data, that were not significant associations in cell-type-specific ca/eQTL data. We used the qvalue() function with lambda parameters equal to seq(0.2,0.8,0.1) from the qvalue R package [97] to estimate *π*_0_ value, and calculated *π*_1_ which is equal to 1 − *π*_0_.

To assess replication of mediation analysis, we extracted variant-methylation site-gene triplets in ROSMAP/xQTL data, if CIT* p*-value [59] for causal model (pCausal or pCausalM) was lower than a *p*-value threshold (0.05/m = 2.39 × 10^−6^, where m is the number of tested triplets) and *p*-value for reactive model (pReactive or pReactiveM) was higher than same *p*-value threshold as described in the ROSMAP/xQTL study [43]. If a variant in ROSMAP/xQTL mediation data was in LD (LD *r*^2^ > 0.8 computed by using our study population) with another variant in the cell-type-specific mediation results, we considered these two variants as the same locus.

### Replication of cell-type-specific trans-eQTL data in fetal bulk brain

To assess replication, we also compared our cell-type-specific trans-eQTL data to trans-eQTLs performed in fetal bulk tissue with a higher sample size (*N*_donor_ = 235) [15]. To this end, we tested the association of variants significantly associated with cis-eGenes detected in our previous study [14] with the other non-pseudogenes across the genome by using the following model where a random effect was included with covariance equal to the kinship matrix K multiplied by the genetic variance:

$$\mathrm{Fetal}\;\mathrm{bulk}\;\mathrm{brain}\;\mathrm{eQTL}\;:\;\mathrm{expression}\sim\mathrm{distal}\;\mathrm{SNP}+10\;\mathrm{MDS}\;\mathrm{of}\;\mathrm{global}\;\mathrm{genotype}+10\;\mathrm{PCs}\;\mathrm{of}\;\mathrm{global}\;\mathrm{gene}\;\mathrm{expression}$$

To evaluate the overlap between two datasets, we applied *π*_1_ statistics via the qvalue R package with lambda parameters equal to seq(0.1,max(*p*-values),0.05) (*p*-values correspond to list of *p*-values from bulk trans-eQTL data) [97]. We estimated that the fraction of significant progenitor and neuron eSNP-trans (downstream) gene pairs that are non-null associations in fetal bulk trans-eQTL data (*π*_1_) by using corresponding *p*-values SNP-Gene pairs detectable in both datasets (*n*_SNP-Gene pairs_ = 34 in progenitors and *n*_SNP-Gene pairs_ = 36 in neurons).

### GWAS co-localization analysis

To find eQTLs colocalized with index GWAS loci, we performed LD-thresholded colocalization analysis for each cell type separately [98]. We used summary statistics from GWAS for schizophrenia (SCZ) [2], major depression disorder (MDD) [99], educational attainment (EA) [6], and cortical thickness and surface area from ENIGMA project [5], UKBB [4], bipolar disorder (BP) [100], neuroticism [101], IQ [102], cognitive performance (CP) [6], attention-deficit/hyperactivity disorder (ADHD) [103], Alzheimer’s disease (AD) [104], and Parkinson’s disease (PD) [105]. We defined index GWAS SNPs where two LD-independent GWAS signals so as to have pairwise LD *r*^2^ < 0.2 based on LD matrix computed with the European population of 1000 Genomes (1000G European phase 3) at genome-wide significant threshold *p*-value (5 × 10^–8^). Then, we found (1) two highly correlated variants (pairwise LD *r*^2^ between GWAS and QTL index variant was higher than 0.8 based on either our study or European population), and (2) if the gene expression/chromatin accessibility was no longer significantly associated with QTL index variant upon conditioning on GWAS index variant, these two loci were considered co-localized.

### Cross-mappability of genes

We used a pre-computed multi-mapping score from a previous study [96]. For each upstream–downstream gene pair, we used symmetric cross-mappability between upstream gene (gene A) and downstream gene (gene B) that was calculated as (crossmap(A,B) + crossmap(B,A))/2 [96]. We discarded upstream–downstream gene pairs if the cross-mappability scores between them were higher than 5 at the log2 scale.

### Transcription factor motif analysis

We used motif breaker R to detect the disruption of the transcription motif binding site where there was a variant within a chromatin accessibility peak [57]. To detect transcription motifs within gene promoters, we used TFBStools [94] with 80% minimum matching score by searching a target sequence within + / − 500 bp window from gene TSS.

## Supplementary Information

**Additional file 1:** **Figure S1.** Colocalized ca/eQTLs and hyperparameter selection for bmediatR. a) The number of variants, chromatin accessible regions and gene expression tested as candidate X-M-Y triplets. b) Proportion of shared and unshared candidate X-M-Ytriplets based on the directionality of the genetic effect for each cell-type. c) Sum of marginal joint likelihood at different PVE_A, PVE_B and PVE_C hyperparameters at odds scale. **Figure S2.** ca/eQTLs at *CHL1* and *FOXD4L1* loci for each cell type. a) Phenotypevs genotype boxplots per cell-type. b) Association of the residualized *CHL1* expression by technical covariates and chromatin accessibility at the peak harboring the variant residualized by technical covariates with the chromatin accessibility at peak1or peak2residualized by technical covariates. c) Phenotypevs genotype boxplots per cell-type. **Figure S3.** Comparison of bayesian and regression-based mediation analyses. a) Comparison of X-M-Y triplets supporting causal forward model detected by regression-based vs bmediatR method in progenitorsand neurons. b) Comparison of X-M-Y triplets supporting causal forward model detected by regression-based vs bmediatR method in progenitors after they were downsampled. **Figure S4.** Replication of cell-type-specific ca/eQTL data in xQTL ROSMAP data. a) Replication of cell-type-specific caQTLsand eQTLsin ROSMAP/xQTL DNA methylation QTL and eQTL data via *π*1 statistics. *π*1 was estimated for ROSMAP mQTL/eQTL data corresponding to primary caQTL/eQTL datawas estimated for randomly sampled ROSMAP mQTL/eQTL data. The error bars represent 95% confidence intervals upon bootstrapping of p-values. b) Number of overlaps between cell-type-specific mediation analysis and ROSMAP xQTL mediation analysis. c) Genomic tracks illustrating association of the variants with DNA methylation and *DNAJC15* expression in ROSMAP data, chromatin accessibility and *DNAJC15* expression in progenitors, and chromatin accessibility and *DNAJC15* expression in neurons. Data points were colored based on the pairwise LD r2 with the rs17553284. The dashed lines indicate p-value threshold for significance in each dataset. d) Coverage plot illustrating ATAC-seq reads within the chromatin accessible region per genotype. The genomic position of the DNA methylation site and rs17553284 were shown. The right diagram illustrates the relationship between rs17553284 and molecular phenotypes. e) Mediation analysis results for ROSMAP, progenitor and neuron data at the locus. CIT p-values at -log10 scale for ROSMAP/xQTL data, and bmediatR posterior probabilities for cell-type-specific data per model are given on the y-axis. Posterior probabilities of causal reactive were set to zero by bmediatR since reactive model priors were not evaluated for mediation via chromatin accessibility. **Figure S5.** Measurement error differences between ATAC-seq and RNA-seq and detection of false positive reactive models. a) Intraclass correlation coefficientfor ATAC-seq measured peaks and RNA-seq measured genes in progenitors and in neurons. Unpaired t-test p-values were shown. b) Simulation analysis for model flipping from causal forward to causal reactive given the error term on mediator. The impact of ICC, mediated heritability and heritability of mediator values on model flipping. Posterior probability of each model was indicated by different colored lines. c) Depiction of the algorithm used to eliminate false positive reactive results at a low threshold ICC value. **Figure S6.** Evaluation of causal reactive model at *DBX2* locus. a) Genotype vs phenotype boxplots for *DBX2* expression and chromatin accessibility in progenitors. b) Location of chromatin accessibility within *DBX2* gene body, and coverage plot for chromatin accessibility across genotypes. c) Mediation scan plot illustrating causal reactive model whereby only *DBX2* gene expression leads to chromatin accessibility, but not any other genes encoding TFs with matching motifs within chromatin accessible region. d) Another variant, rs2731038, within the chromatin accessible region that was in LD with rs2731040, showed allele-specific-chromatin accessibility. Padj: Adjusted *p*-value after ASCA analysis.

**Additional file 2:** **Table S1.** Causal forward and reactive mediation results for ca/eQTL colocalizations, and replicated data with ROSMAP/xQTL study.

**Additional file 3:** **Table S2.** Causal forward and reactive mediation results for cis/trans eQTL colocalizations.

**Additional file 4:** **Table S3.** Colocalization of ca/eSNPs with GWAS.

**Additional file 5. **Review history.

## Data Availability

Code used to conduct analyses in this manuscript is available at https://bitbucket.org/steinlabunc/pathqtl/src/master/. A copy of this repository is also available in Zenodo https://doi.org/10.5281/zenodo.7819209 [106] under the license Creative Commons Attribution 4.0 International. BAM files for RNA-seq and genotype data are on dbGAP with study accession number phs002493.v1.p1 [107], and BAM file for ATAC-seq data are on dbGAP with study accession number phs001958.v1.p1 [108]. We stored full summary statistics for eQTL, caQTL, trans-eQTL, and cis-eQTL data in Zenodo https://doi.org/10.5281/zenodo.7820257 [109] under license Creative Commons Attribution 4.0 International. We obtained full summary statistics of xQTL data of the ROSMAP study [43] from https://mostafavilab.stat.ubc.ca/xqtl/. We accessed transcription factor binding motifs [93] from http://humantfs.ccbr.utoronto.ca/download.php.
