# Supplementary material for: Inferring cell-type-specific causal gene regulatory networks during human neurogenesis
Source: Genome Biol. 2023 May 30;24:130. doi: 10.1186/s13059-023-02959-0 (PMC10230710; doi:10.1186/s13059-023-02959-0)
Supplement: Supplementary file 1 — Additional file 1: Figure S1. Colocalized ca/eQTLs and hyperparameter selection for bmediatR. a) The number of variants, chromatin accessible regions and gene expression tested as candidate X-M-Y triplets. b) Proportion of shared and unshared candidate X-M-Ytriplets based on the directionality of the genetic effect for each cell-type. c) Sum of marginal joint likelihood at different PVE_A, PVE_B and PVE_C hyperparameters at odds scale. Figure S2. ca/eQTLs at CHL1 and FOXD4L1 loci for each cell type. a) Phenotypevs genotype boxplots per cell-type. b) Association of the residualized CHL1 expression by technical covariates and chromatin accessibility at the peak harboring the variant residualized by technical covariates with the chromatin accessibility at peak1or peak2residualized by technical covariates. c) Phenotypevs genotype boxplots per cell-type. Figure S3. Comparison of bayesian and regression-based mediation analyses. a) Comparison of X-M-Y triplets supporting causal forward model detected by regression-based vs bmediatR method in progenitorsand neurons. b) Comparison of X-M-Y triplets supporting causal forward model detected by regression-based vs bmediatR method in progenitors after they were downsampled. Figure S4. Replication of cell-type-specific ca/eQTL data in xQTL ROSMAP data. a) Replication of cell-type-specific caQTLsand eQTLsin ROSMAP/xQTL DNA methylation QTL and eQTL data via π1 statistics. π1 was estimated for ROSMAP mQTL/eQTL data corresponding to primary caQTL/eQTL datawas estimated for randomly sampled ROSMAP mQTL/eQTL data. The error bars represent 95% confidence intervals upon bootstrapping of p-values. b) Number of overlaps between cell-type-specific mediation analysis and ROSMAP xQTL mediation analysis. c) Genomic tracks illustrating association of the variants with DNA methylation and DNAJC15 expression in ROSMAP data, chromatin accessibility and DNAJC15 expression in progenitors, and chromatin accessibility and DNAJC15 expression in neuron [file 13059_2023_2959_MOESM1_ESM.pdf]

Fig. S1

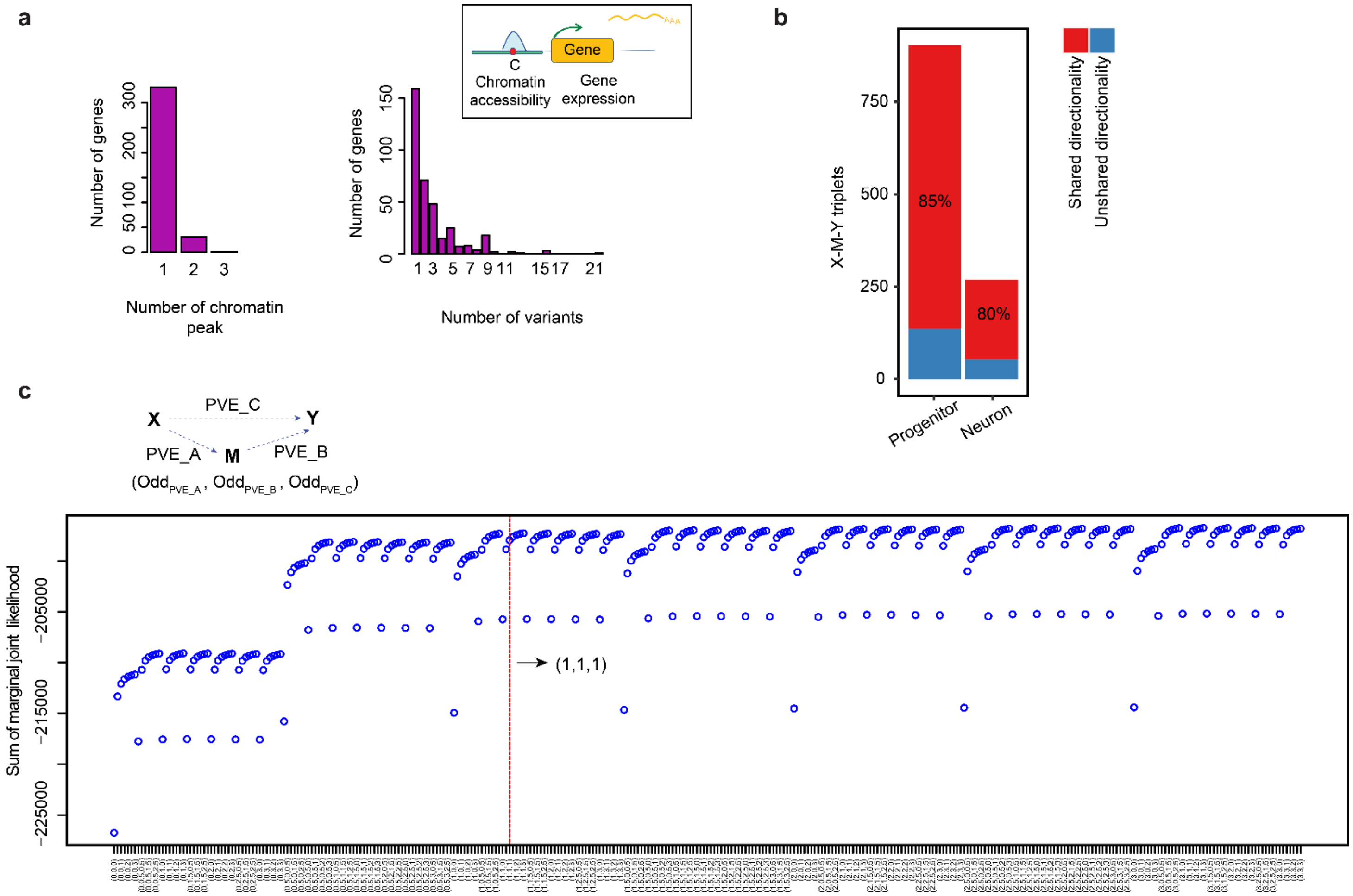

Figure S1. Colocalized ca/eQTLs and hyperparameter selection for bmediatR.

a) The number of variants, chromatin accessible regions and gene expression tested as candidate X-M-Y triplets.

b) Proportion of shared and unshared candidate X (genetic variant)-M (chromatin accessibility)-Y (gene expression) triplets based on the directionality of the genetic effect for each cell-type.

c) Sum of marginal joint likelihood at different PVE_A, PVE_B and PVE_C hyperparameters at odds scale.

Fig. S2

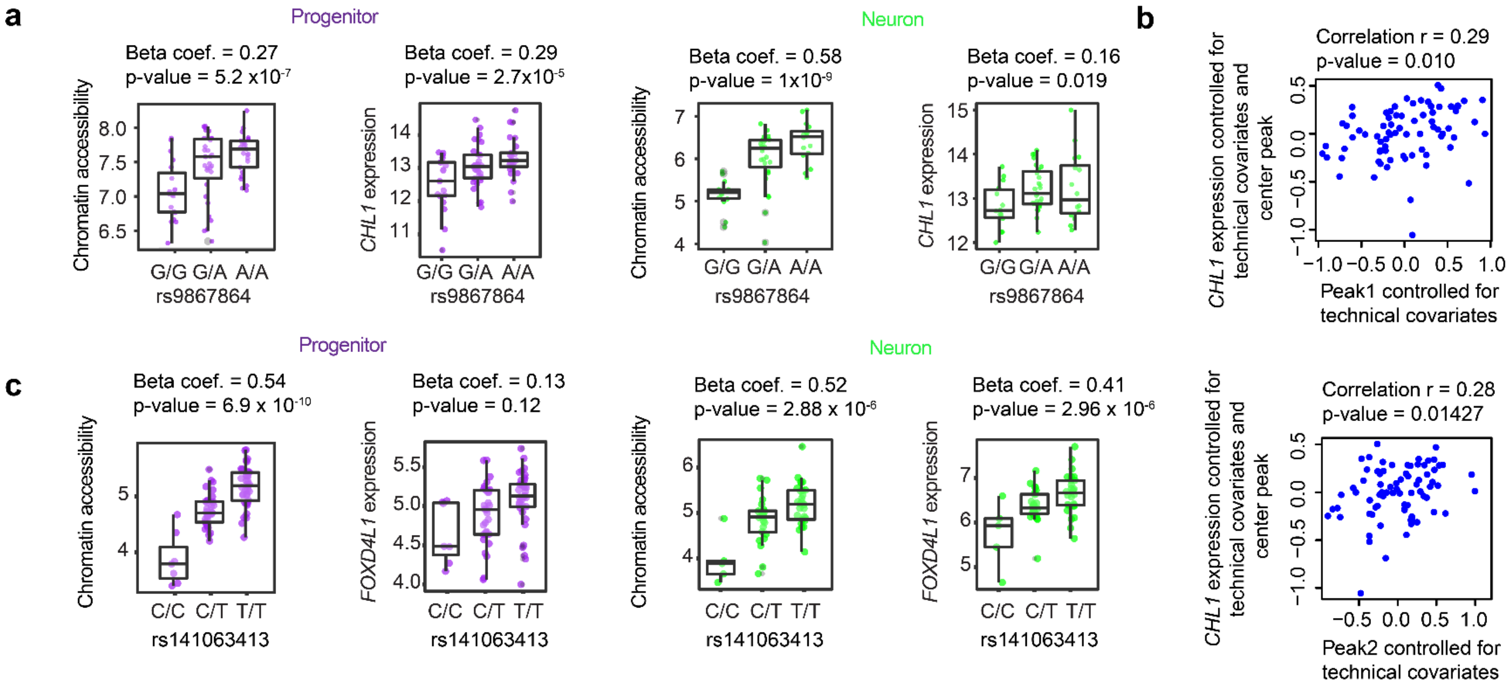

Figure S2. ca/eQTLs at *CHL1* and *FOXD4L1* loci for each cell type.

- **a)** Phenotype (chromatin accessibility and *CHL1* expression) vs genotype boxplots per cell-type.
- **b)** Association of the residualized *CHL1* expression by technical covariates and chromatin accessibility at the peak harboring the variant residualized by technical covariates with the chromatin accessibility at peak1 (upper plot) or peak2 (lower plot) residualized by technical covariates
- **c)** Phenotype (chromatin accessibility and *FOXD4L1* expression) vs genotype boxplots per cell-type.

Fig. S3

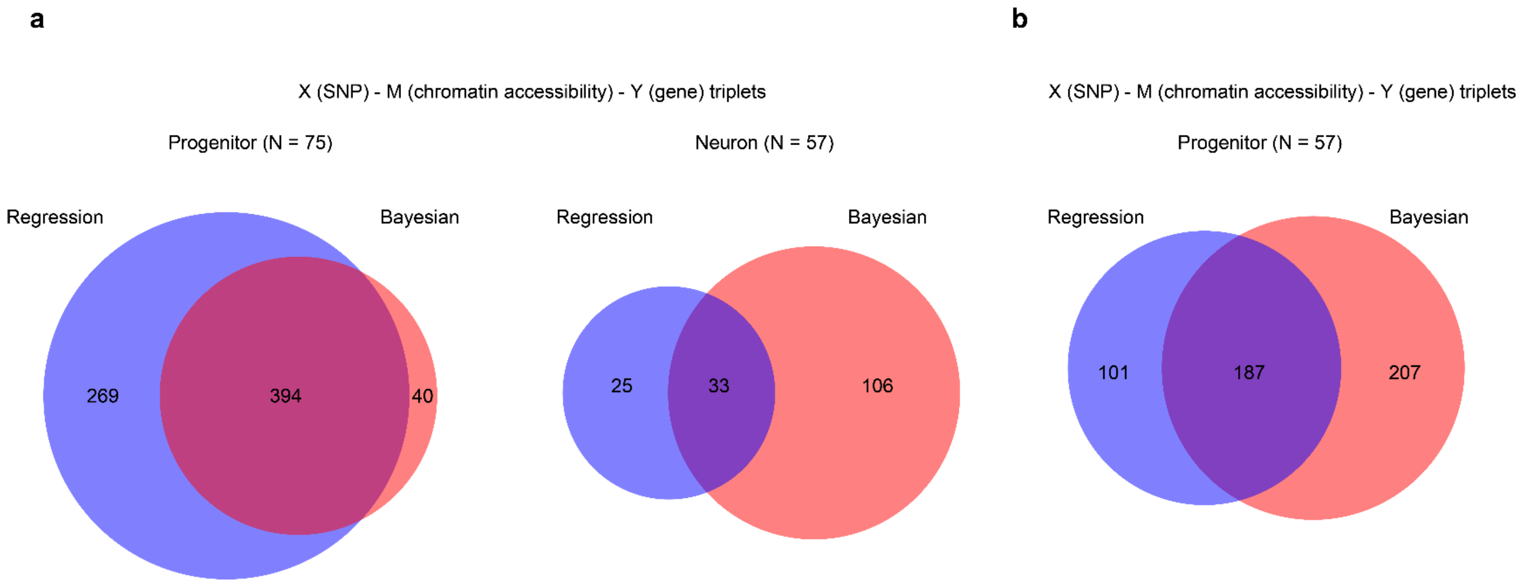

Figure S3. Comparison of bayesian and regression-based mediation analyses.

a) Comparison of X-M-Y triplets supporting causal forward model detected by regression-based vs bmediatR method in progenitors (N_{donor} = 75) and neurons (N_{donor} = 57).

b) Comparison of X-M-Y triplets supporting causal forward model detected by regression-based vs bmediatR method in progenitors after they were downsampled (N_{donor} = 57).

Fig. S4

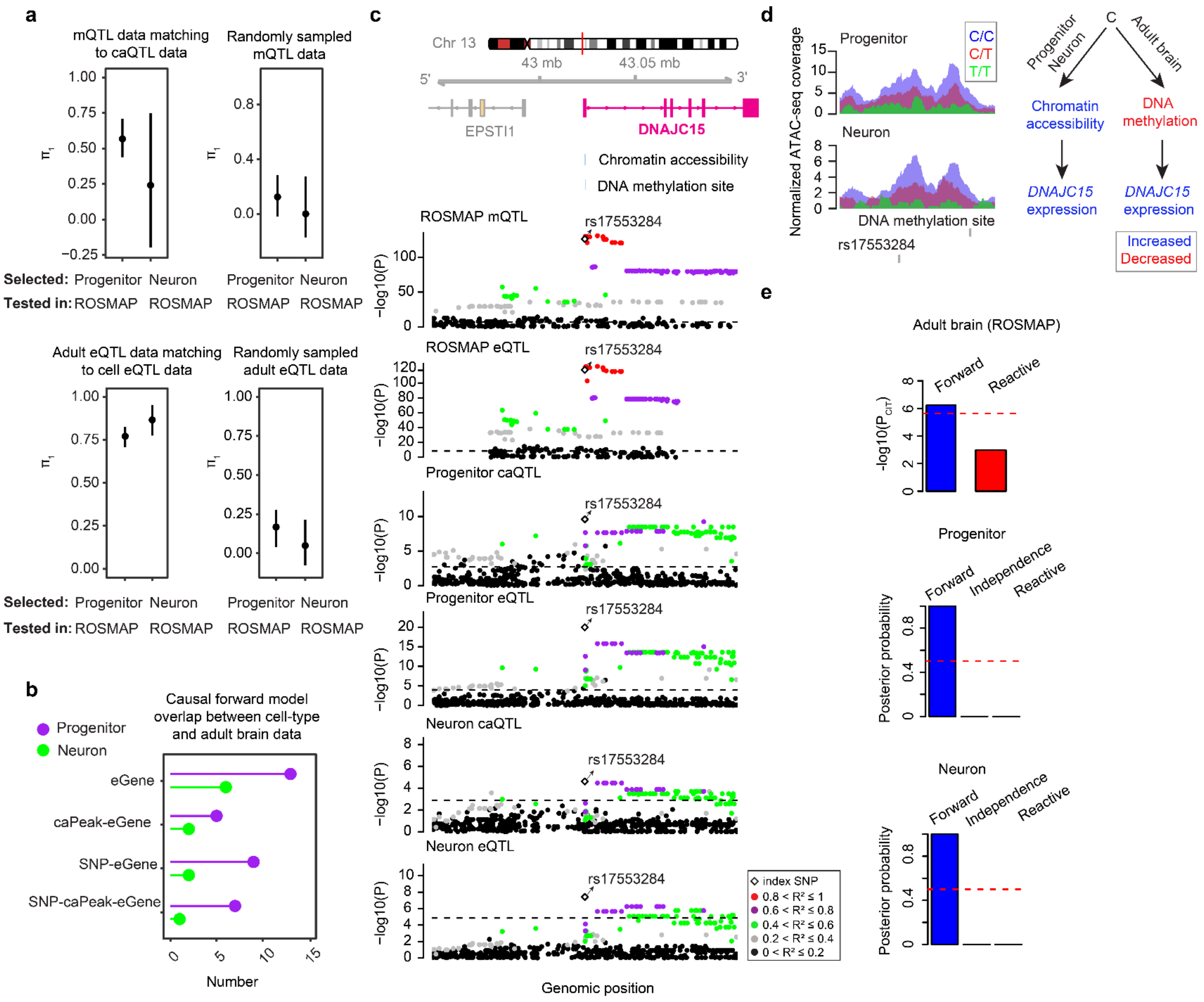

Figure S4. Replication of cell-type-specific ca/eQTL data in xQTL ROSMAP data.

a) Replication of cell-type-specific caQTLs (upper) and eQTLs (lower) in ROSMAP/xQTL DNA methylation QTL and eQTL data via π_1 statistics. π_1 was estimated for ROSMAP mQTL/eQTL data corresponding to primary caQTL/eQTL data (left) was estimated for randomly sampled ROSMAP mQTL/eQTL data (right). The error bars represent 95% confidence intervals upon bootstrapping of p-values.

b) Number of overlaps between cell-type-specific mediation analysis and ROSMAP xQTL mediation analysis.

c) Genomic tracks illustrating association of the variants with DNA methylation and *DNAJC15* expression in ROSMAP data, chromatin accessibility and *DNAJC15* expression in progenitors, and chromatin accessibility and *DNAJC15* expression in neurons. Data points were colored based on the pairwise LD r^2 with the rs17553284. The dashed lines indicate p-value threshold for significance in each dataset.

d) Coverage plot illustrating ATAC-seq reads within the chromatin accessible region per genotype. The genomic position of the DNA methylation site and rs17553284 were shown (Left side). The right diagram illustrates the relationship between rs17553284 and molecular phenotypes.

e) Mediation analysis results for ROSMAP, progenitor and neuron data at the locus. CIT p-values at -log10 scale for ROSMAP/xQTL data, and bmediatR posterior probabilities for cell-type-specific data per model are given on the y-axis. Posterior probabilities of causal reactive were set to zero by bmediatR since reactive model priors were not evaluated for mediation via chromatin accessibility.

Fig. S5

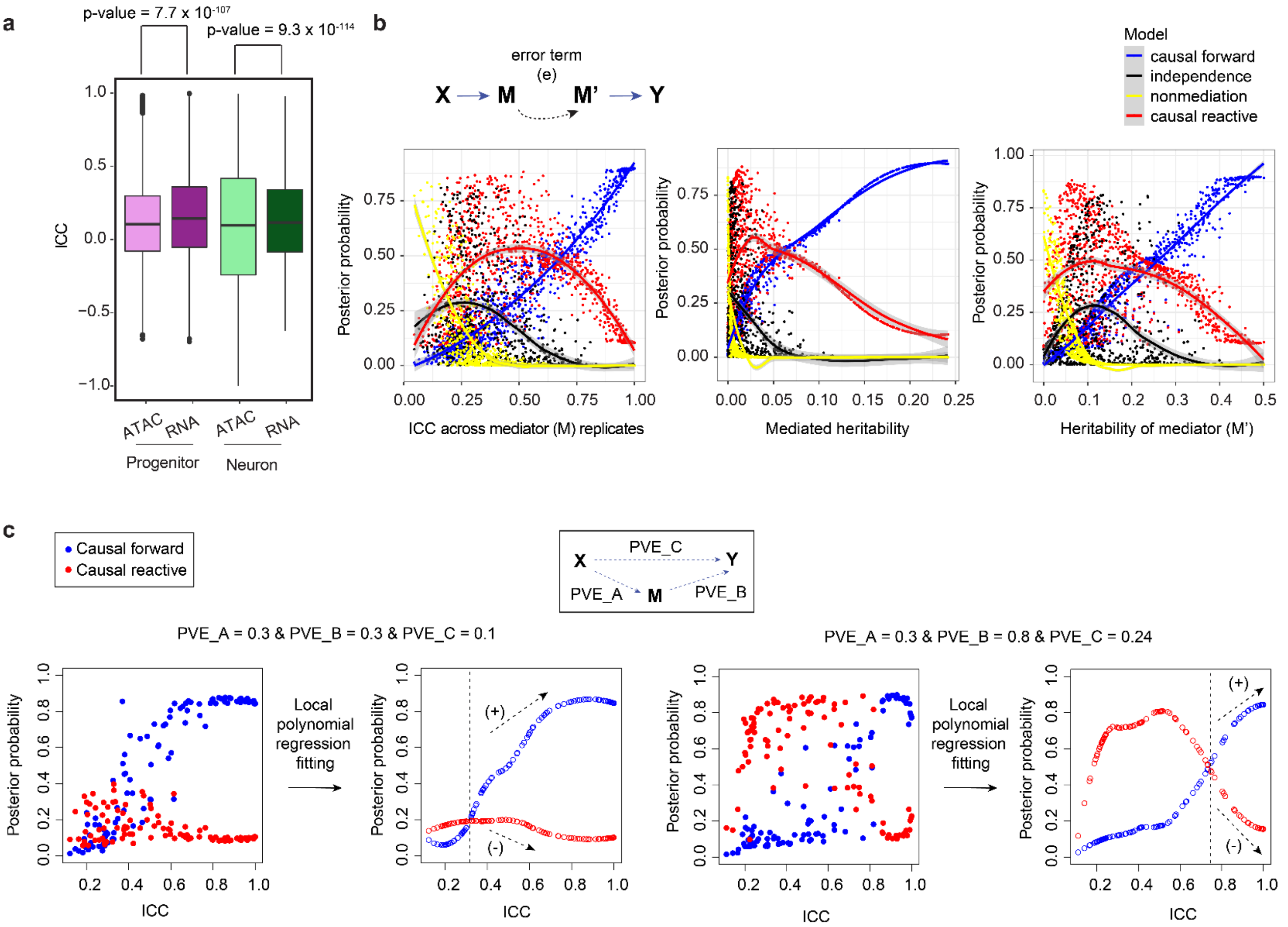

Figure S5. Measurement error differences between ATAC-seq and RNA-seq and detection of false positive reactive models.

- **a)** Intraclass correlation coefficient (ICC) for ATAC-seq measured peaks and RNA-seq measured genes in progenitors and in neurons. Unpaired t-test p-values were shown.
- **b)** Simulation analysis for model flipping from causal forward to causal reactive given the error term on mediator (M). The impact of ICC, mediated heritability and heritability of mediator values on model flipping. Posterior probability of each model was indicated by different colored lines.
- **c)** Depiction of the algorithm used to eliminate false positive reactive results at a low threshold ICC value.

Fig. S6

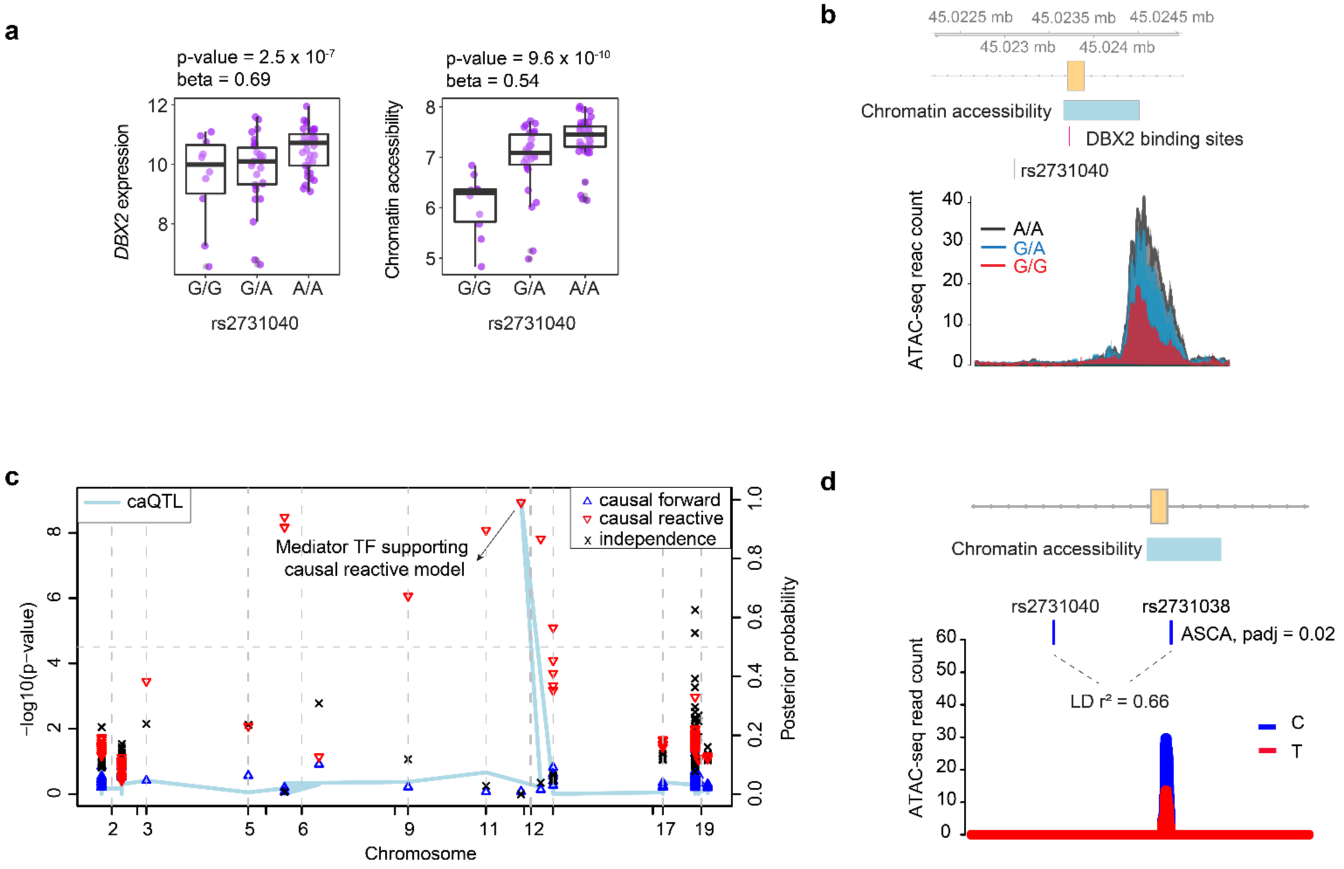

Figure S6. Evaluation of causal reactive model at *DBX2* locus.

a) Genotype vs phenotype boxplots for *DBX2* expression and chromatin accessibility in progenitors.

b) Location of chromatin accessibility within *DBX2* gene body, and coverage plot for chromatin accessibility across genotypes.

c) Mediation scan plot illustrating causal reactive model whereby only *DBX2* gene expression leads to chromatin accessibility, but not any other genes encoding TFs with matching motifs within chromatin accessible region.

d) Another variant, rs2731038, within the chromatin accessible region that was in LD with rs2731040 (r^2 = 0.66), showed allele-specific-chromatin accessibility (ASCA). Padj: Adjusted p-value after ASCA analysis.
