# Supplementary material for: Inferring cell-type-specific causal gene regulatory networks during human neurogenesis
Source: Genome Biol. 2023 May 30;24:130. doi: 10.1186/s13059-023-02959-0 (PMC10230710; doi:10.1186/s13059-023-02959-0)
Supplement: Supplementary file 5 — Additional file 5. Review history. [file 13059_2023_2959_MOESM5_ESM.docx]

Review History

**First round of review**

**Reviewer 1**

**Were you able to assess all statistics in the manuscript, including the appropriateness of statistical tests used?**

Yes, and I have assessed the statistics in my report.

**Comments to author:**

The authors perform statistical mediation analyses to identify causal relationship between triples of SNPs, chromatin accessibility regions, and gene expression. They use SNP genotype data, ATAC-Seq, and RNA-Seq from the same individuals in two cell types (n=75 progenitors and b=57 neurons). Three specific analysis are done for each of the cell types. First, statistical mediation analysis is performed to identify instances where the relationship between a SNP (S) genotype and gene expression levels (G) is mediated by chromatin accessibility (M); i.e., S->M-> G vs S->G->M vs independence of the effect. Next, similar type of mediation analysis is used to find trans-eQTLs vs a eGene mediates the relationship between a distal SNP and gene expression. Finally, the results are analyzed together with some relevant GWAS studies.

Although the research questions are timely and important to the field, I found the analysis preliminary, lacking robust new insights, with limited context for the observed results. I will summarize major comments below.

- Sample size for performing eQTL analysis is relatively small. Previous studies (e.g., Battle et al., GR 2013) have shown in sub-sampling analysis that low hundreds of samples are needed for truly informative cis-eQTL discovery, and thousands of samples for trans-eQTL analysis. The limited sample size in this study limits the interpretability of the results. For example, the authors notes that ~23% of mediation events found by one statistical approach (Bayesian model) agree with another model on the same data for neurons. Careful analysis shows that sample size is a major issue for lack of concordance. Given such low concordance on the same data but different method limits any interpretation one drives from the neuron sample.
- There are no replication results reported. This is critical for understanding the generalizability of the results. As cited by the authors, much larger mediation analysis exist for brain tissue (e.g., Ng et al., Nature Neuro 2017). Why the authors didn't perform replication analysis for eQTL and mediation analysis? (while DNAm and ATAC-Seq are different, perhaps some replication in previous data will provide additional support.)
- Previous mediation analysis seem to indicate a lower number of truly causally mediated events (e.g., 10% in Ng et al., 2017). I wonder if low sample sizes here are inflating the results.
- I find the trans-eQTL results problematic. Several studies have highlighted artifacts in trans-eQTLT analysis with low sample sizes (summarized in Saha et al., 2019). The authors have investigated one type of artifact ("homology"), however, a much more careful replication analysis is needed for the discovery, including adjustments for known and hidden confounding factors. The standard threshold in trans-eQTL studies is typically more stringent that what authors used here (e.g., 10-8 in Yap et al., 2018; 10-12 in Battle et al.) and also, replication analysis is needed given the larger number of trans-eQTLs reported here, compared to previous studies with larger samples.

**Reviewer 2**

**Were you able to assess all statistics in the manuscript, including the appropriateness of statistical tests used?**

Yes, and I have assessed the statistics in my report.

**Comments to author:**

Overall
The authors describe an application of the recently proposed bmediatR Bayesian model section mediation analysis approach to identify genetic variation in progenitors and neurons that affects gene expression in local and distal genes, mediated by chromatin accessibility. The topic is important and relevant, the paper is well written, and the analysis is complex but appears to be well thought-out and executed. A limitation of the article is that key details are purportedly included in references rather than described in the main text. For example, the eigenMT-FDR method is used for QTL detection but not explained.

Specific Comments
Lines starting on 448. Section: Cell-type-specific eQTL and caQTL mapping to test causal forward model. More details on the QTL modeling strategy are needed. For example, how many variants were tested in each tissue, what was the test statistic and what were the discovery thresholds? How were the MDS and kinship values calculated?

The number of covariates in the QTL models seems high relative the sample sizes. For the neurons, 1 for SNP + 10 for MDS + 1 for FACS (maybe more?) + 7 PCs = 19 total for only 57 observations. That's only 3 observations per covariate, which is generally thought to be over-fitting. Please justify.

Line 466. eigenMT-FDR method for QTL detection should be explained even if briefly so readers are not required to read other articles to understand the basics of what was done.

Lines starting on 212. Section: Accuracy of Bayesian mediation approach in classifying forward versus reactive models given differences in measurement error between phenotypes
I found this to be an interesting and unique approach for assessing the effects of measurement error on the causal inferences.

Lines starting on 422. Section: Generation of cell-type-specific ATAC-seq and RNA-seq data
What was read depth? What does it mean that ATACseq was done with HiSeq2500 "or" MiSeq platforms? Were both used or is it unknown which was used?

Lines starting on 429. Section: Genotype processing and imputation
"Genotyping was performed using Illumina HumanOmni2.5 or HumanOmni2.5Exome platforms." It is not clear what "or" means here. Please describe in more detail.

**Authors Response**

**Point-by-point responses to the reviewers’ comments:**

**We thank both reviewers for their very helpful comments and suggestions. Overall, the reviewers had positive sentiments about our work, stating that:**

Reviewer 1 : “*the research questions are timely and important to the field*”

Reviewer 2: “*The topic is important and relevant, the paper is well written, and the analysis is complex but appears to be well thought-out and executed*” and *“I found this to be an interesting and unique approach for assessing the effects of measurement error on the causal inferences.”*

**We appreciate the extensive critiques and have addressed all of them in detail, resulting in substantial improvements in our manuscript. The major new analyses performed in response to reviewer concerns were:**

1. **We compared our cell-type-specific chromatin accessibility and expression QTL datasets to xQTL data from the ROSMAP study, as suggested by Reviewer 1, finding evidence for the replication of our results.**
2. **We re-performed the trans-eQTL analysis by increasing the minor allele frequency threshold, eliminating pseudogenes, and applying multiple testing correction to address the concerns of Reviewer 1. We also performed trans-eQTL analysis in higher sample size fetal bulk brain data to evaluate replication of our trans-eQTL analysis. The number of trans-eQTLs dramatically reduced upon these adjustments.**
3. **We evaluated if the number of covariates utilized in QTL analyses led to overfitting issues to address the concern of reviewer 2. To this end, we reduced the number of genotype PCs from 10 to 3, and compared the overlap in significant associations, which yielded a high overlapping rate.**

**In addition to addressing the reviewers’ concerns, we also made several text corrections, and fixed Figures 3d and 3e and the position of the line corresponding to the significance threshold for caQTL genomic tracks in Figure 5a, which was mistakenly based on the eQTL threshold. After these updates, the results presented in Figure 3e became significant in progenitors, and the conclusion drawn from Figure 3d and Figure 5a did not change.**

**Below are our point-by-point replies to each question in bold:**

Reviewer #1: The authors perform statistical mediation analyses to identify causal relationship between triples of SNPs, chromatin accessibility regions, and gene expression. They use SNP genotype data, ATAC-Seq, and RNA-Seq from the same individuals in two cell types (n=75 progenitors and b=57 neurons). Three specific analysis are done for each of the cell types. First, statistical mediation analysis is performed to identify instances where the relationship between a SNP (S) genotype and gene expression levels (G) is mediated by chromatin accessibility (M); i.e., S->M-> G vs S->G->M vs independence of the effect. Next, similar type of mediation analysis is used to find trans-eQTLs vs a eGene mediates the relationship between a distal SNP and gene expression. Finally, the results are analyzed together with some relevant GWAS studies.

Although the research questions are timely and important to the field, I found the analysis preliminary, lacking robust new insights, with limited context for the observed results. I will summarize major comments below.

**We thank the reviewer for their positive appraisal of the importance of our work. We have modified our manuscript in response to the reviewer’s concern to replicate our cell-type-specific data with higher powered datasets, and to conduct trans-eQTL analysis with more conventional thresholds. Our analyses performed in response to the reviewer’s concerns have confirmed the robustness of our findings. We believe that these analyses provide additional support to our conclusions of temporal and cell-type eQTL specificity with insights into GWAS loci.**

-	Sample size for performing eQTL analysis is relatively small. Previous studies (e.g., Battle et al., GR 2013) have shown in sub-sampling analysis that low hundreds of samples are needed for truly informative cis-eQTL discovery, and thousands of samples for trans-eQTL analysis. The limited sample size in this study limits the interpretability of the results. For example, the authors notes that ~23% of mediation events found by one statistical approach (Bayesian model) agree with another model on the same data for neurons. Careful analysis shows that sample size is a major issue for lack of concordance. Given such low concordance on the same data but different method limits any interpretation one drives from the neuron sample.

**We appreciate the reviewer’s concern regarding the small sample size of neuron QTLs. Though sample sizes used here are smaller than current bulk eQTL studies, the dataset used still represents the largest currently available human cell-type specific progenitor and neuron e/caQTL dataset. Previous studies investigating the regulatory mechanism of the variants associated with neuropsychiatric disease risk and inter-individual differences in other brain-relevant traits were mainly performed in the bulk adult brain tissue** [[1,2]**.**](https://paperpile.com/c/PLu3DK/XwyL+yPM8) **However, brain-trait associated variants are also enriched within regulatory elements of neural progenitors and early born neurons that are present in the developing human brain, but not in the adult brain** [[3,4]**.**](https://paperpile.com/c/PLu3DK/h624+5Th57) **The discovery of developmental specific eQTLs in the bulk fetal brain demonstrated this developmental specificity; however, the heterogeneity of bulk tissue may mask cell-type-specific allelic effects** [[5,6]**.**](https://paperpile.com/c/PLu3DK/lycv+gsE2) **Also, there are a very limited number of studies that generated different molecular QTLs derived from the same individuals to allow mediation analysis. In this study, we addressed all of these challenges, which**

**resulted in a limited sample size because: (1) to establish a cell-type-specific system, we cultured the neural progenitors and differentiated them into neurons, and the cell lines from some donors died during differentiation; (2) to perform mediation analysis, we needed to utilize donors for which both ATAC-seq and RNA-seq data were available. The requirement for overlap between two molecular phenotypes also reduced the sample size, given that there were cases in which either of the sequencing data from the same donors did not pass quality checks. Hence, there was a tradeoff between higher sample size vs cell-type-specificity and integration of different molecular phenotypes in our study. However, despite a decrease in power, we still were able to make cell-type specific causal inferences that we now show**

**(below) can be replicated in other brain datasets. Nevertheless, we acknowledge the limitation of our sample size in the discussion.**

**The relevant text is copied and modified text is underlined below:**

**“Although our sample size was comparable with other cell-based QTL studies** [[7–10]**,**](https://paperpile.com/c/PLu3DK/7E2YQ+nKDQg+S7zUA+MgcEX) **increasing the number of donors will likely lead to a higher number of shared QTLs enabling a more systematic and higher powered mediation analysis.”**

-	There are no replication results reported. This is critical for understanding the generalizability of the results. As cited by the authors, much larger mediation analysis exist for brain tissue (e.g., Ng et al., Nature Neuro 2017). Why the authors didn’t perform replication analysis for eQTL and mediation analysis? (while DNAm and ATAC-Seq are different, perhaps some replication in previous data will provide additional support.)

**We performed two replication analyses suggested by the reviewer. First, we compared our cell-type-specific caQTL and eQTL data with the methylation and eQTL data from the adult brain in the ROSMAP/xQTL dataset** [[11]**.**](https://paperpile.com/c/PLu3DK/xQZB) **Since the statistical power is different between datasets, we applied *n-*1 statistics for comparison** [[12]**.**](https://paperpile.com/c/PLu3DK/Dsrf) **To compare our cell-type-specific caQTL data with adult brain methylation QTL data, we initially found methylation sites or CpG islands harboring the methylation sites overlapped with chromatin accessibility peaks in our caQTL data, and defined these as overlapping regulatory regions. We observed that the fraction of progenitor and neuron primary SNP-overlapping regulatory region pairs that are non-null associations in adult brain ROSMAP methylation QTL data (*n-*1) was 56.7% and 24.1% when subsetting to SNP-regulatory region pairs that were detectable in both datasets (Fig. S4a). For the comparison of our cell-type-specific eQTL data with adult brain ROSMAP eQTL data, we observed that the fraction of progenitor and neuron primary eSNP-eGene pairs that are non-null associations in ROSMAP eQTL data (*n-*1) was 77.1% and 86.6% when subsetting to SNP-Gene pairs detectable in both datasets (Fig. S4a). Estimates for sharing of ca/mQTLs and eQTLs across datasets were well above randomly sampled SNPs in ROSMAP/xQTL data, which showed nonsignificant associations in our cell-type-specific data, demonstrating clear evidence for replication of the QTL findings.**

**Next, we compared our mediation analysis results with the mediation analysis performed in Ng et al. study** [[11]**.**](https://paperpile.com/c/PLu3DK/xQZB) **Similar to QTL data comparison, we considered methylation sites or CpG islands harboring the methylation sites overlapped with chromatin accessibility peaks in our caQTL as overlapping epigenetic regulatory regions. We found 7 and 1 SNP-chromatin accessibility peak-eGene triplets in progenitor and neuron showed mediation in causal forward direction in both our analysis and adult brain ROSMAP data. We also observed that 9 and 2 SNP-eGene pairs, 5 and 2 chromatin accessibility peak-eGene pairs, and 13 and 6 eGenes in progenitor and neurons supported the causal forward model in both our and ROSMAP datasets (Fig. S4b). As an example of a mediation locus supported by both datasets, we highlighted a causal regulatory region observed in progenitor, neuron, and**

**ROSMAP data at *DNAJC15* gene locus (Fig. S4c-e). We found that variant rs17553284 within a chromatin accessibility region detected in our cell-type-specific data was a caQTL and eQTL in both progenitor and neuron data, and mQTL and eQTL in ROSMAP data. While the C allele of the variant increased *DNAJC15* expression in progenitor, neuron and bulk adult brain data; the same allele decreased DNA methylation and increased chromatin accessibility in progenitor and neuron data consistent with the expected opposite regulatory direction of DNA methylation and chromatin accessibility. Additionally, causal forward mediation was replicated across all datasets (Fig. S4e).**

**Given the differences in molecular phenotypes, tissue age and heterogeneity, and the algorithm employed for mediation analysis between our cell-type-specific data vs ROSMAP data, we would expect low overlap. However, despite all of these differences, observation of some level of replication supports the generalizability of our results.**

**The relevant figures and text are copied below and modified text is underlined:**

**“Given that sample size may influence causality inferences, we sought to assess replication in another brain tissue dataset where multiple gene regulatory modalities and genotypes were acquired on the same individuals (ROSMAP/xQTL)** [[11]**.**](https://paperpile.com/c/PLu3DK/xQZB) **To assess replication of our cell-type-specific ca/eQTL data in relevant datasets, we computed *n-*1 statistics** [[12]](https://paperpile.com/c/PLu3DK/Dsrf) **within adult brain methylation QTL and eQTL data from the previous study** [[11]](https://paperpile.com/c/PLu3DK/xQZB) **for significant primary variant-chromatin accessibility and variant-Gene pairs. We found that the fraction of progenitor and neuron primary SNP-overlapping epigenetic regulatory region pairs that are non-null associations in adult brain ROSMAP/xQTL methylation QTL data (*n-*1) was 56.7% and 24.1% when subsetting to SNP-epigenetic regulatory region pairs that were detectable in both datasets (Fig. S4a). Also, we observed that the fraction of progenitor and neuron primary eSNP-eGene pairs that are non-null associations in ROSMAP/xQTL eQTL data (*n-*1) was 77.1% and 86.6% when subsetting to SNP-Gene pairs detectable in both datasets (Fig. S4a). Estimates for sharing of ca/mQTLs and eQTLs across datasets were well above randomly sampled SNPs in ROSMAP/xQTL data, that showed nonsignificant associations in our cell-type-specific data, demonstrating clear evidence for replication of the QTL findings.**

**We also compared the mediation analysis results between our study and the ROSMAP/xQTL study** [[11]**.**](https://paperpile.com/c/PLu3DK/xQZB) **We detected 7 and 1 SNP-chromatin accessibility peak-eGene triplets in progenitors and neurons supported mediation in causal forward direction in both our analysis and xQTL study (Fig. S4b). As a specific example, we observed a causal regulatory region detected in progenitor, neuron and ROSMAP/xQTL data at *DNAJC15* gene locus (Fig. S4c). We found that variant rs17553284 within a chromatin accessibility region at gene promoter was a caQTL and an eQTL in both progenitor and neuron data, and a mQTL and an eQTL in ROSMAP/xQTL data. While the C allele of the variant increased *DNAJC15* expression**

**in progenitor, neuron and ROSMAP/xQTL data; it decreased DNA methylation and increased chromatin accessibility in progenitor and neuron data consistent with the opposite regulatory direction of DNA methylation and chromatin accessibility (Fig. S4d). Importantly, the chromatin accessible region detected in our cell-type-specific data mediated *DNAJC15* expression in both progenitor and neurons, also DNA methylation site detected in ROSMAP/xQTL data mediated *DNAJC15* expression (bmediatR posterior probability for causal forward: progenitor = 0.99, neuron = 0.99 and causal inference test (CIT)** [[13]](https://paperpile.com/c/PLu3DK/HUjkm) **p-value in xQTL for causal forward = 5.9 x 10^-7^ and for causal reactive = 0.001, Fig. S4e). Despite differences in sample age, heterogeneity and method used for mediation analysis, observing the replication between two datasets suggested our approach is robust. ”**

**“To assess the replication of our ca/eQTL data in the ROSMAP/xQTL data** [[11]**,**](https://paperpile.com/c/PLu3DK/xQZB) **we initially liftovered the positions of methylation sites and variants from hg19 to hg38. Within this dataset, we detected methylation sites or CpG islands harboring the methylation sites (obtained from Illumina HumanMethylation450 manifest file) overlapped with chromatin accessibility peaks in our caQTL data, and defined these as overlapping regulatory regions. We estimated the fraction of progenitor and neuron primary SNP-overlapping regulatory region pairs that are non-null associations in adult brain ROSMAP methylation QTL data by using the corresponding p-values to SNP-regulatory region pairs that were detectable in both datasets via *n-*1 statistics** [[12]**.**](https://paperpile.com/c/PLu3DK/Dsrf) **For the comparison of our cell-type-specific eQTL data with adult brain ROSMAP eQTL data, we estimated the fraction of progenitor and neuron primary eSNP-eGene pairs that are non-null associations in ROSMAP eQTL data (*n-*1) by using the corresponding p-values to SNP-Gene pairs detectable in both datasets. We also randomly sampled nonsignificant SNP-regulatory region pairs or SNP-gene pairs in ROSMAP data to demonstrate that the *n-*1 value calculated via non-null associations was not by chance. We used the qvalue() function with lambda parameters equal to seq(0.2,0.8,0.1) from the R package qvalue** [[14]](https://paperpile.com/c/PLu3DK/cL17B) **to estimate *n-*0 value, and calculated *n-*1 which is equal to 1 - *n-*0.**

**To assess replication of mediation analysis, we extracted variant-methylation site-gene triplets in ROSMAP/xQTL data, if CIT p-value** [[13]](https://paperpile.com/c/PLu3DK/HUjkm) **for causal model (pCausal or pCausalM) was lower than a p-value threshold (0.05/m = 2.39 x 10^-6^, where m is the number of tested triplets) and p-value for reactive model (pReactive or pReactiveM) was higher than same p-value threshold as described in the ROSMAP/xQTL study** [[11]**.**](https://paperpile.com/c/PLu3DK/xQZB) **If a variant in ROSMAP/xQTL mediation data was in LD (LD r^2^ > 0.8 computed by using our study population) with another variant in the cell-type-specific mediation results, we considered these two variants as the same locus.”**

Figure S4

-	Previous mediation analysis seem to indicate a lower number of truly causally mediated events (e.g., 10% in Ng et al., 2017). I wonder if low sample sizes here are inflating the results.

**Comparing the percentages of mediated events across these two studies is difficult. We restricted the variants tested for mediation to be within chromatin accessible regions to evaluate a more biologically plausible causal regulatory mechanism (681 and 204 variants were tested in progenitors and neurons in our data, of which 364 and 87 supported the causal forward model). On the other hand, Ng et al., 2017 included variants within 5 kb of methylation sites (10,897 variants were tested in Ng et al., 2017 of which ~980 variants supported the causal model). We expect that our variants within peaks are more likely to be causal, increasing chances for discovering causal mediation, and resulting in a higher proportion of variants supporting the causal model. We believe the replication analyses suggested by the reviewer in the comment**

**above give greater credence to our discoveries, yet we still acknowledge our low sample size as described in the discussion.**

-	I find the trans-eQTL results problematic. Several studies have highlighted artifacts in

trans-eQTLT analysis with low sample sizes (summarized in Saha et al., 2019). The authors have investigated one type of artifact (“homology”), however, a much more careful

replication analysis is needed for the discovery, including adjustments for known and hidden confounding factors. The standard threshold in trans-eQTL studies is typically more stringent that what authors used here (e.g., 10-8 in Yap et al., 2018; 10-12 in Battle et al.) and also, replication analysis is needed given the larger number of trans-eQTLs reported here, compared to previous studies with larger samples.

**To address the reviewer’s concern, we adjusted the trans-eQTL analysis by adapting the following modifications either from Battle et al.** [[15]](https://paperpile.com/c/PLu3DK/5uLh) **or GTEx Consortium** [[16]](https://paperpile.com/c/PLu3DK/HinP) **: (1) We only included variants with minor allele frequency greater than 0.025 and primary SNPs associated with cis genes and pruned variants based on LD r^2^ > 0.5. (2) We excluded any cis- or trans- pseudogenes. (3) We applied a multiple testing correction and defined significant trans-eQTL at 10% FDR. As the reviewer also noted, we already considered and excluded artifacts caused by homology in the original manuscript, and for the revised manuscript, we again eliminated any upstream-downstream gene pairs whose log2(cross-mappability) score was greater than 5 prior to computing multiple testing correction. We controlled for unknown confounding factors in the previous submission by adding principal components (PCs) of global gene expression. These numbers were selected since they maximized the number of eGenes during cis-eQTL mapping. We utilized PCs given their higher performance compared to other hidden variable inference methods to detect cis and trans-eQTLs** [[17]**.**](https://paperpile.com/c/PLu3DK/u24t)

**After these modifications, the number of trans-eQTLs was dramatically reduced: we detected 35 and 30 variants cis-regulating 23 and 17 upstream genes and also associated with 22 and 21 downstream genes in progenitor and neurons, respectively (at 10% FDR).**

**To assess replication, we also compared our cell-type-specific trans-eQTL data to trans-eQTLs performed in fetal bulk tissue with a higher sample size (Ndonor = 235)** [[6]**.**](https://paperpile.com/c/PLu3DK/gsE2) **To this end, we tested the association of variants significantly associated with cis-eGenes detected in our previous study** [[18]](https://paperpile.com/c/PLu3DK/Ua5IF) **with the other non-pseudogenes across the genome. To evaluate the overlap between two datasets, we applied *π*1 statistics. We estimated that the fraction of significant progenitor and neuron eSNP-trans (downstream) gene pairs that are non-null associations in fetal bulk trans eQTL data (*π*1) was 95.5% and 74.2% when subsetting to SNP-Gene pairs detectable in both datasets (nSNP-Gene pairs = 34 in progenitors and nSNP-Gene pairs = 36 in neurons). This provides strong evidence of replication of the trans-eQTL results.**

**Out of these trans-eQTLs, we observed that 11 and 12 downstream genes were mediated through 13 and 10 upstream genes regulated by 23 and 22 variants in progenitor and neurons, respectively. Accordingly, Figure 4 was updated, and Figure 6 and Table S3 second and third sheets were removed. Also, we decided not to include the analysis (Figure S6) comparing bmediatR results to MOSTWAS, since low number of detected trans-Genes would lead to an inaccurate comparison.**

**The relevant figures and text are copied below and modified text is underlined:**

**“To perform trans-eQTL analysis, we first selected a list of variants with significant evidence of cis-regulation for at least one gene in our previous analysis (Ndonor = 85 in progenitors and Ndonor = 74 in neurons)** [[18]**.**](https://paperpile.com/c/PLu3DK/Ua5IF) **We further included variants with minor allele frequency greater than 0.025, and included primary SNPs associated with cis genes and pruned variants via PLINK v.1.90b3 software** [[19]](https://paperpile.com/c/PLu3DK/3AAR3) **(parameters --indep-pairwise 50 5 0.5). We excluded cis- or trans- pseudogenes, and any upstream-downstream gene pairs if they had a cross-mappability value** [[20]](https://paperpile.com/c/PLu3DK/48TUV) **greater than 5 at log2 scale. For trans-eQTL analysis, we included variant-gene pairs if the distance between variant and TSS of the gene was larger than 1MB on the same chromosomes or if the variant was on a different chromosome than the gene. We tested associations of the filtered variants with all the filtered genes in the genome that were expressed in our dataset. To conduct trans-eQTL analysis, we utilized EMMAX software** [[21]](https://paperpile.com/c/PLu3DK/VAj4T) **controlling for population structure and unknown technical confounders by adding number of PCs of global expression maximizing eGene discovery in our previous cis-eQTL analysis** [[18]**.**](https://paperpile.com/c/PLu3DK/Ua5IF) **The models used for trans-eQTL analysis per cell-type were:**

**Progenitor eQTL: expression - distal SNP + 10 MDS of global genotype + 10 PCs of global gene expression + kinship matrix**

**Neuron eQTL: expression - distal SNP + 10 MDS of global genotype + FACS sorter + 12 PCs of global gene expression + kinship matrix**

**To detect significant trans-eQTLs, we performed multiple testing correction, and defined the associations as significant at 10% FDR. ”**

**“To assess replication, we also compared our cell-type-specific trans-eQTL data to trans-eQTLs performed in fetal bulk tissue with a higher sample size (Ndonor = 235)** [[6]**.**](https://paperpile.com/c/PLu3DK/gsE2) **To this end, we tested the association of variants significantly associated with cis-eGenes detected in our previous study** [[18]](https://paperpile.com/c/PLu3DK/Ua5IF) **with the other non-pseudogenes across the genome by using the following model:**

**Fetal bulk brain eQTL: expression - distal SNP + 10 MDS of global genotype + kinship matrix + 10 PCs of global gene expression**

**To evaluate the overlap between two datasets, we applied *π*1 statistics via the qvalue R package with lambda parameters equal to seq(0.1,max(p-values),0.05) (p-values**

**correspond to list of p-values from bulk trans-eQTL data)** [[14]**.**](https://paperpile.com/c/PLu3DK/cL17B) **We estimated that the fraction of significant progenitor and neuron eSNP-trans (downstream) gene pairs that are non-null associations in fetal bulk trans eQTL data (*n-*1) by using corresponding p-values SNP-Gene pairs detectable in both datasets (nSNP-Gene pairs = 34 in progenitors and nSNP-Gene pairs = 36 in neurons).”**

**“Within each cell-type, SNPs with significant association to a proximal gene (cis-eSNPs, primary SNPs per gene as well as pruned SNPs, see Methods) were also tested for association with every other gene (trans-eQTL, see Methods). At a 10% FDR significance threshold, we detected 35 and 30 variants cis-regulating 23 and 17 upstream genes and also associated with 22 and 21 downstream genes in progenitor and neurons, respectively. We assessed whether cell-type-specific trans-eQTL results were replicated in fetal bulk brain data (Ndonor = 235)** [[6,18]**.**](https://paperpile.com/c/PLu3DK/Ua5IF+gsE2) **The fraction of significant progenitor and neuron eSNP-trans (downstream) gene pairs that are non-null associations in fetal bulk trans eQTL data (*n-*1) was estimated to be 95.5% and 74.2% when subsetting to SNP-Gene pairs detectable in both datasets, providing strong evidence for replication. After performing mediation analysis, we discovered 11 and 12 downstream genes that were mediated via trans-regulation by 13 and 10 cis-regulated upstream genes in progenitors and neurons, respectively (Fig. 4a; posterior probability for causal forward > 0.5, Table S2).”**

**“As an example, *XPO1* on chromosome 2, was cis-regulated by a progenitor-specific eSNP (rs138286618) and the same variant was associated with the expression of genes *PTBP1* on chromosome 19 and *BBX* on chromosome 3 (Fig. 4b and 4c). *PTBP1* and *XPO1* genes were positively correlated; whereas *BBX* and *XPO1* genes were negatively correlated (Fig. 4d). The mediation analysis showed that the genetic effects of the variant on *PTBP1* and *BBX* were mediated via *XPO1* gene expression (posterior probabilities supporting causal forward = 0.99 and 0.99 for both *PTBP1* and *BBX,* Fig. 4e). XPO1, which is a nuclear export protein** [[22]**,**](https://paperpile.com/c/PLu3DK/7BNCZ) **induced apoptosis of cortical neural progenitors in mice** [[23]](https://paperpile.com/c/PLu3DK/bvPG6) **and intronic mutations within XPO1 were associated with autism** [[23,24]**.**](https://paperpile.com/c/PLu3DK/bvPG6+OD9Ol) **PTBP1 protein is an RNA-binding protein, which was upregulated in glioblastoma** [[25]](https://paperpile.com/c/PLu3DK/ZRw08) **and had a role in the regulation of neuronal differentiation** [[26]**.**](https://paperpile.com/c/PLu3DK/nlUoY) **BBX protein is a transcription factor which is highly expressed in progenitor cells of developing neocortex and found as a downstream target of NFIX transcription factor regulating neural development** [[27]**.**](https://paperpile.com/c/PLu3DK/Tnhwk) ***XPO1* may therefore represent an important master regulator of disease associated genes in progenitors during neural development.”**

**Figure 4**

**Reviewer #2:** Overall

The authors describe an application of the recently proposed bmediatR Bayesian model section mediation analysis approach to identify genetic variation in progenitors and neurons that affects gene expression in local and distal genes, mediated by chromatin accessibility. The topic is important and relevant, the paper is well written, and the analysis is complex but appears to be well thought-out and executed. A limitation of the article is that key details are purportedly included in references rather than described in the main text. For example, the eigenMT-FDR method is used for QTL detection but not explained.

**We thank the reviewer for their positive appraisal of our work. To make the paper easier to follow we added more details and clarifications in the methods section. Additionally, we evaluated if the number of covariates used during QTL analysis caused overfitting.**

Specific Comments

Lines starting on 448. Section: Cell-type-specific eQTL and caQTL mapping to test causal forward model. More details on the QTL modeling strategy are needed. For example, how many variants were tested in each tissue, what was the test statistic and what were the discovery thresholds? How were the MDS and kinship values calculated?

**We adjusted the relevant section as follows where modifications are underlined:**

**“To find the candidate SNP-chromatin accessibility-gene candidates to be tested for causal forward model or independence, we tested association of 171,256 and 161,716 variants located within chromatin accessible regions and within +/- 1MB of gene transcription sites both with 55,473 and 54,044 chromatin accessibility regions via caQTL analysis, and with 22,710 and 22,696 genes via eQTL analysis in progenitor and neurons, respectively. To perform QTL analyses, we utilized a linear mixed effects regression model via EMMAX software** [[21]](https://paperpile.com/c/PLu3DK/VAj4T) **in which we controlled for population stratification (ancestry) and cryptic relatedness. To control for population structure, we computed the first 10 MDS components of ancestry for all the individuals with genotype information available via PLINK software with (parameters: –cluster –mds-plot 10). To control cryptic relatedness, we generated kinship matrix from non-imputed genotype data via emmax-kin -v -h -d algorithm in EMMAX software, in which we excluded the genetic variants on the same chromosome with the tested variants in QTL analysis** [[28]**.**](https://paperpile.com/c/PLu3DK/bIawU) **We controlled for unknown technical confounders by using the same number of PCs of global chromatin accessibility and gene expression models as previously established for caQTLs** [[4]](https://paperpile.com/c/PLu3DK/5Th57) **and eQTLs** [[18]](https://paperpile.com/c/PLu3DK/Ua5IF) **for each cell-type. In these studies, the number of PCs was determined as the number maximizing caQTL discovery for caQTL analysis at 5% FDR and as the number maximizing eGene discovery at 5% FDR for eQTL analysis by sequentially adding PCs into covariate matrix. The models were used as follows:”**

The number of covariates in the QTL models seems high relative the sample sizes. For the neurons, 1 for SNP + 10 for MDS + 1 for FACS (maybe more?) + 7 PCs = 19 total for only

57 observations. That’s only 3 observations per covariate, which is generally thought to be over-fitting. Please justify.

**We compared ca/eQTL results by reducing the number of MDS for ancestry from 10 to 3 to answer the reviewer’s concern. The significant associations from these two analyses were highly consistent. We observed that 93.8%/92.2% of caPeaks and 90.2%/88% of primary caPeak-caSNP pairs discovered via controlling for 10 MDS ancestry components were also discovered when we controlled for 3 MDS ancestry components. Also, we observed that 92.4%/91.4% of eGenes and 83.1%/79.6% of primary eGene-eSNP pairs discovered via controlling for 10 MDS ancestry components were also discovered when we controlled for 3 ancestry components. The number of eGenes/caPeaks was higher when we controlled for 3 MDS components instead of 10, indicating that our approach was conservative.**

**To determine how many principal components (PCs) to include for global chromatin accessibility and gene expression QTLs, we applied an optimization strategy in our previous studies** [[4,18]](https://paperpile.com/c/PLu3DK/5Th57+Ua5IF) **that is a widely used procedure in the field** [[29,30]**.**](https://paperpile.com/c/PLu3DK/lYxP+cC9m) **During this procedure, we sequentially added PCs of global chromatin accessibility/expression into the covariate matrix and calculated the number of caQTLs and eGenes that survived the 5% FDR threshold. We chose 8 and 7 PCs of global chromatin accessibility in progenitors and neurons since it maximized the discovery of caQTLs, and 10 and 12 PCs of global gene expression which maximized the discovery of eGenes.**

**The relevant figures and text are copied below and modified text is underlined:**

**“We controlled for unknown technical confounders by using the same number of PCs of global chromatin accessibility and gene expression models as previously established for caQTLs** [[4]](https://paperpile.com/c/PLu3DK/5Th57) **and eQTLs** [[18]](https://paperpile.com/c/PLu3DK/Ua5IF) **for each cell-type. In these studies, the number of PCs was determined as the number maximizing caQTL discovery for caQTL analysis at 5% FDR and as the number maximizing eGene discovery at 5% FDR for eQTL analysis by sequentially adding PCs into covariate matrix.”**

Response to reviewer Figure 1

Line 466. eigenMT-FDR method for QTL detection should be explained even if briefly so readers are not required to read other articles to understand the basics of what was done.

**We described the eigenMT-FDR method as the reviewer requested. The modified text was underlined as below:**

**“We applied the same hierarchical multiple testing correction strategy as previously by computing a global eigenMT-FDR p-value after local adjustment per eGene** [[18]**.**](https://paperpile.com/c/PLu3DK/Ua5IF) **To perform eigenMT-FDR procedure** [[31]**,**](https://paperpile.com/c/PLu3DK/2htRO) **briefly, (1) nominal p-values of all cis variants per gene were locally adjusted via the eigenMT method** [[32]**.**](https://paperpile.com/c/PLu3DK/afn1R) **(2) We performed multiple testing correction by using these locally adjusted p-values from the top SNPs per gene, which resulted in globally adjusted p-values. (3) Genes with globally adjusted p value lower than 0.05 were defined as eGenes. For caQTLs, we adjusted association p-values via only the Benjamini-Hochberg method given that there were a few chromatin accessible regions with multiple variants, and retained the associations with lower than 5% FDR. ”**

Lines starting on 212. Section: Accuracy of Bayesian mediation approach in classifying forward versus reactive models given differences in measurement error between phenotypes

I found this to be an interesting and unique approach for assessing the effects of measurement error on the causal inferences.

**We thank the reviewer for their positive comment.**

Lines starting on 422. Section: Generation of cell-type-specific ATAC-seq and RNA-seq data

What was read depth? What does it mean that ATACseq was done with HiSeq2500 “or” MiSeq platforms? Were both used or is it unknown which was used?

**ATAC-seq and RNA-seq data had an average read depth of 25.5 million and 99.8 million read pairs. We sequenced some of the ATAC-seq libraries with HiSeq2500 platform, and some of them with MiSeq platform.**

**The relevant text is copied below and modified text is underlined:**

**“As previously described** [[4]**,**](https://paperpile.com/c/PLu3DK/5Th57) **we prepared ATAC-seq libraries and sequenced some of the libraries by using Illumina HiSeq2500 and some of them via MiSeq platforms with 50 bp paired-end sequencing with an average depth of 25.5 million read pairs. We aligned them to the human genome (GRCh38/hg38) by reducing mapping bias via WASP method after quality control as described previously** [[4]**.**](https://paperpile.com/c/PLu3DK/5Th57) **We generated RNA-seq libraries, performed sequencing with NovaSeq S2 flow cell using 150 bp paired-end sequencing with an average read depth of 99.8 million read pairs, and also mapped them to the human genome (GRCh38/hg38) after quality control as previously described** [[18]**.**](https://paperpile.com/c/PLu3DK/Ua5IF)**”**

Lines starting on 429. Section: Genotype processing and imputation

“Genotyping was performed using Illumina HumanOmni2.5 or HumanOmni2.5Exome platforms.” It is not clear what “or” means here. Please describe in more detail.

**We used Illumina HumanOmni2.5 platform for the genotyping of some of the DNA samples, and HumanOmni2.5Exome platform for genotyping others. These platforms are almost identical, though the HumanOmni2.5Exome includes some exonic variants in addition to the variants designed to tag the entirety of the genome.**

**The relevant text is copied below and modified text is underlined:**

**“We performed genotyping of some of the DNA libraries via Illumina HumanOmni2.5 and some of them via HumanOmni2.5Exome platforms.”**

References

1. [Li M, Santpere G, Imamura Kawasawa Y, Evgrafov OV, Gulden FO, Pochareddy S, et al. Integrative functional genomic analysis of human brain development and neuropsychiatric risks. Science [Internet]. 2018;362. Available from:](http://paperpile.com/b/PLu3DK/XwyL) <http://dx.doi.org/10.1126/science.aat7615>
2. [Fromer M, Roussos P, Sieberts SK, Johnson JS, Kavanagh DH, Perumal TM, et al. Gene expression elucidates functional impact of polygenic risk for schizophrenia. Nat Neurosci. 2016;19:1442–53.](http://paperpile.com/b/PLu3DK/yPM8)
3. [de la Torre-Ubieta L, Stein JL, Won H, Opland CK, Liang D, Lu D, et al. The Dynamic Landscape of Open Chromatin during Human Cortical Neurogenesis. Cell. 2018;172:289– 304.e18.](http://paperpile.com/b/PLu3DK/h624)
4. [Liang D, Elwell AL, Aygün N, Krupa O, Wolter JM, Kyere FA, et al. Cell-type-specific effects of genetic variation on chromatin accessibility during human neuronal differentiation. Nat Neurosci. 2021;24:941–53.](http://paperpile.com/b/PLu3DK/5Th57)
5. [O’Brien HE, Hannon E, Hill MJ, Toste CC, Robertson MJ, Morgan JE, et al. Expression quantitative trait loci in the developing human brain and their enrichment in neuropsychiatric disorders. Genome Biol. 2018;19:194.](http://paperpile.com/b/PLu3DK/lycv)
6. [Walker RL, Ramaswami G, Hartl C, Mancuso N, Gandal MJ, de la Torre-Ubieta L, et al. Genetic Control of Expression and Splicing in Developing Human Brain Informs Disease Mechanisms [Internet]. Cell. 2020. p. 745. Available from:](http://paperpile.com/b/PLu3DK/gsE2) <http://dx.doi.org/10.1016/j.cell.2020.04.016>
7. [Cuomo ASE, Seaton DD, McCarthy DJ, Martinez I, Bonder MJ, Garcia-Bernardo J, et al. Single-cell RNA-sequencing of differentiating iPS cells reveals dynamic genetic effects on gene expression. Nat Commun. 2020;11:810.](http://paperpile.com/b/PLu3DK/7E2YQ)
8. [Alasoo K, Rodrigues J, Mukhopadhyay S, Knights AJ, Mann AL, Kundu K, et al. Shared genetic effects on chromatin and gene expression indicate a role for enhancer priming in immune response. Nat Genet. 2018;50:424–31.](http://paperpile.com/b/PLu3DK/nKDQg)
9. [Jerber J, Seaton DD, Cuomo ASE, Kumasaka N, Haldane J, Steer J, et al. Population-scale single-cell RNA-seq profiling across dopaminergic neuron differentiation. Nat Genet. 2021;53:304–12.](http://paperpile.com/b/PLu3DK/S7zUA)
4. [Pickrell JK, Marioni JC, Pai AA, Degner JF, Engelhardt BE, Nkadori E, et al. Understanding mechanisms underlying human gene expression variation with RNA sequencing. Nature. 2010;464:768–72.](http://paperpile.com/b/PLu3DK/MgcEX)
5. [Ng B, White CC, Klein H-U, Sieberts SK, McCabe C, Patrick E, et al. An xQTL map integrates the genetic architecture of the human brain’s transcriptome and epigenome. Nat Neurosci. 2017;20:1418–26.](http://paperpile.com/b/PLu3DK/xQZB)
6. [Storey JD, Tibshirani R. Statistical significance for genomewide studies. Proc Natl Acad Sci U S A. 2003;100:9440–5.](http://paperpile.com/b/PLu3DK/Dsrf)
7. [Millstein J, Zhang B, Zhu J, Schadt EE. Disentangling molecular relationships with a causal inference test. BMC Genet. 2009;10:23.](http://paperpile.com/b/PLu3DK/HUjkm)
8. [Dabney A, Storey JD, Warnes GR. qvalue: Q-value estimation for false discovery rate control. R package version [Internet]. 2010;1. Available from:](http://paperpile.com/b/PLu3DK/cL17B) <ftp://ftp.uni-bayreuth.de/pub/math/statlib/R/CRAN/src/contrib/Descriptions/qvalue.html>
9. [Battle A, Mostafavi S, Zhu X, Potash JB, Weissman MM, McCormick C, et al. Characterizing the genetic basis of transcriptome diversity through RNA-sequencing of 922 individuals. Genome Res. 2014;24:14–24.](http://paperpile.com/b/PLu3DK/5uLh)
10. [The GTEx Consortium. The Genotype-Tissue Expression (GTEx) pilot analysis: Multitissue gene regulation in humans. Science. American Association for the Advancement of Science; 2015;348:648–60.](http://paperpile.com/b/PLu3DK/HinP)
11. [Zhou HJ, Li L, Li Y, Li W, Li JJ. PCA outperforms popular hidden variable inference methods for molecular QTL mapping. Genome Biol. 2022;23:210.](http://paperpile.com/b/PLu3DK/u24t)
12. [Aygün N, Elwell AL, Liang D, Lafferty MJ, Cheek KE, Courtney KP, et al. Brain-trait-associated variants impact cell-type-specific gene regulation during neurogenesis. Am J Hum Genet. 2021;108:1647–68.](http://paperpile.com/b/PLu3DK/Ua5IF)
13. [Chang CC, Chow CC, Tellier LC, Vattikuti S, Purcell SM, Lee JJ. Second-generation PLINK: rising to the challenge of larger and richer datasets. Gigascience. 2015;4:7.](http://paperpile.com/b/PLu3DK/3AAR3)
14. [Saha A, Battle A. False positives in trans-eQTL and co-expression analyses arising from RNA-sequencing alignment errors. F1000Res. 2018;7:1860.](http://paperpile.com/b/PLu3DK/48TUV)
15. [Kang HM, Sul JH, Service SK, Zaitlen NA, Kong S-Y, Freimer NB, et al. Variance component model to account for sample structure in genome-wide association studies. Nat Genet. 2010;42:348–54.](http://paperpile.com/b/PLu3DK/VAj4T)
16. [Azmi AS, Uddin MH, Mohammad RM. The nuclear export protein XPO1 — from biology to targeted therapy [Internet]. Nature Reviews Clinical Oncology. 2021. p. 152–69. Available from:](http://paperpile.com/b/PLu3DK/7BNCZ) <http://dx.doi.org/10.1038/s41571-020-00442-4>
17. [Li X, Feng Y, Yan M, Tu X, Xie B, Ni F, et al. Inhibition of Autism-Related Crm1 Disrupts Mitosis and Induces Apoptosis of the Cortical Neural Progenitors. Cereb Cortex. 2020;30:3960–76.](http://paperpile.com/b/PLu3DK/bvPG6)
18. [Liu X, Malenfant P, Reesor C, Lee A, Hudson ML, Harvard C, et al. 2p15–p16.1 microdeletion syndrome: molecular characterization and association of the OTX1 and XPO1 genes with autism spectrum disorders [Internet]. European Journal of Human Genetics. 2011. p. 1264–70. Available from:](http://paperpile.com/b/PLu3DK/OD9Ol) <http://dx.doi.org/10.1038/ejhg.2011.112>
19. [McCutcheon IE, Hentschel SJ, Fuller GN, Jin W, Cote GJ. Expression of the splicing regulator polypyrimidine tract-binding protein in normal and neoplastic brain. Neuro Oncol. 2004;6:9–14.](http://paperpile.com/b/PLu3DK/ZRw08)
20. [Linares AJ, Lin C-H, Damianov A, Adams KL, Novitch BG, Black DL. The splicing regulator PTBP1 controls the activity of the transcription factor Pbx1 during neuronal differentiation. Elife. 2015;4:e09268.](http://paperpile.com/b/PLu3DK/nlUoY)
21. [Dixon C, Harvey TJ, Smith AG, Gronostajski RM, Bailey TL, Piper M. Nuclear factor one X regulates Bobby sox during development of the mouse forebrain. Cell Mol Neurobiol. 2013;33:867–73.](http://paperpile.com/b/PLu3DK/Tnhwk)
22. [Yang J, Zaitlen NA, Goddard ME, Visscher PM, Price AL. Advantages and pitfalls in the application of mixed-model association methods. Nat Genet. 2014;46:100–6.](http://paperpile.com/b/PLu3DK/bIawU)
23. [GTEx Consortium. The GTEx Consortium atlas of genetic regulatory effects across human tissues. Science. 2020;369:1318–30.](http://paperpile.com/b/PLu3DK/lYxP)
24. [Currin KW, Erdos MR, Narisu N, Rai V, Vadlamudi S, Perrin HJ, et al. Genetic effects on](http://paperpile.com/b/PLu3DK/cC9m)

[liver chromatin accessibility identify disease regulatory variants. Am J Hum Genet. 2021;108:1169–89.](http://paperpile.com/b/PLu3DK/cC9m)

25. [Huang QQ, Ritchie SC, Brozynska M, Inouye M. Power, false discovery rate and Winner’s Curse in eQTL studies [Internet]. Nucleic Acids Research. 2018. p. e133–e133. Available from:](http://paperpile.com/b/PLu3DK/2htRO) <http://dx.doi.org/10.1093/nar/gky780>
26. [Davis JR, Fresard L, Knowles DA, Pala M, Bustamante CD, Battle A, et al. An Efficient Multiple-Testing Adjustment for eQTL Studies that Accounts for Linkage Disequilibrium between Variants. Am J Hum Genet. 2016;98:216–24.](http://paperpile.com/b/PLu3DK/afn1R)

**Second round of review**

**Reviewer 1**

The authors have answered my questions/comments, thanks.

**Reviewer 2**

The authors have adequately addressed my concerns.
